# Environmental Fate of Emerging Contaminants and Their Transformation Products in Mexican Aquatic Systems: Distribution, Persistence, Monitoring Gaps, and Removal

**DOI:** 10.3390/molecules31152574

**Published:** 2026-07-23

**Authors:** Sheila Genoveva Pérez Bravo, Ana Carolina Anzures Mendoza, Ana Lilia Avilés Mariño, Rosa Elena Zamudio Alemán, Dalia Guadalupe Mendoza López, María del Refugio Castañeda Chávez

**Affiliations:** 1 Tecnológico Nacional de México/Instituto Tecnológico de Boca del Río, Carretera Veracruz-Córdoba, km 12, Boca del Río C.P. 94290, Veracruz, Mexico; sheilaperez@bdelrio.tecnm.mx (S.G.P.B.); rosazamudio@bdelrio.tecnm.mx (R.E.Z.A.); dalia.ml@bdelrio.tecnm.mx (D.G.M.L.); 2 Universidad Tecnológica de Altamira, Boulevard de los Ríos km 7, Altamira C.P. 89603, Tamaulipas, Mexico; amendoza@utaltamira.edu.mx; 3 Tecnológico Nacional de México/Instituto Tecnológico de Altamira, Carretera Tampico-Mante, km 24.5, Altamira C.P. 89600, Tamaulipas, Mexico; ana.am@altamira.tecnm.mx

**Keywords:** emerging contaminants, Mexico, water bodies, groundwater, sediments, wastewater treatment plants

## Abstract

Emerging contaminants (ECs) represent a growing environmental problem due to their presence in various environmental matrices. In Mexico, research on ECs remains limited. This scoping review, conducted in accordance with the PRISMA-ScR guidelines, analyzes their environmental fate during the period 2004–2025, considering their distribution in surface and groundwater, sediments, and influents and effluents from wastewater treatment plants (WWTPs). The reviewed studies demonstrate the presence of pharmaceutically active compounds (PhACs), endocrine-disrupting compounds (EDCs), personal care products, phthalates, bisphenols, pesticides, illicit drugs, xanthines, perfluoroalkyl and polyfluoroalkyl substances (PFAS), metabolites, and industrial compounds. The Apatlaco, Cuautla, and Santa Catarina rivers exhibit the greatest diversity of ECs, while groundwater shows evidence of infiltration of PhACs, phthalates, bisphenols, and triclosan, associated with urban, industrial, and tourist discharges. In sediments, PhACs and EDCs accumulate at concentrations in the ng/g range, while phthalates reach concentrations of thousands of ng/g, indicating their affinity for the solid phase. At Mexican wastewater treatment plants, conventional treatment methods do not completely remove ECs, allowing residual amounts to be discharged into receiving bodies of water. Overall, national monitoring should be expanded to include metabolites, transformation products, and advanced treatment assessment.

## 1. Introduction

In recent years, the presence of emerging contaminants (ECs) in surface water bodies has been documented with increasing frequency. In this review, ECs are defined as natural or synthetic chemical compounds that were not previously regulated or systematically monitored, and whose presence, persistence, transformation, or potential effects on the aquatic environment raise scientific, environmental, or health concerns. This group includes endocrine-disrupting compounds (EDCs), personal care products, pharmaceutical active compounds (PhACs), pesticides, illicit drugs, and industrial additives. These compounds are commonly detected in concentrations ranging from ng/L to μg/L; therefore, technically, they can also be considered microcontaminants. Their ubiquity in aquatic environments is primarily due to the fact that conventional wastewater treatment plants (WWTPs) were not designed to efficiently remove these complex chemical structures [1,2]. Currently, most of these compounds are not specifically regulated under Mexican law, which limits strict control over their discharges and systematic environmental monitoring.

Exposure of aquatic organisms to ECs results in various toxic effects that compromise ecosystem health. For example, significant growth inhibition of the microalga *Pseudokirchneriella subcapitata* has been observed following exposure to commonly used PhACs such as acetaminophen (PCM) and ibuprofen (IBU), with IC20-72h inhibition values of 12.7 and 23.9 mg/L, respectively [3]. Similarly, in the macrophyte *Lemna gibba*, diclofenac (DCF) and IBU cause a reduction in the number of fronds, separation of colonies, and chlorosis; for these last two effects, effective concentrations (EC50-168h) of 114.1 and 56.9 mg/L were reported for DCF, respectively, and 171.3 and 126.7 mg/L for IBU, respectively. In invertebrates such as *Daphnia magna*, acute effects of mortality and immobilization have been reported, with LC50-24h values of 132.5 mg/L for DCF and 137.0 mg/L for IBU, while the absence of mobility showed EC50-24h values of 95.4 and 105.6 mg/L for DCF and IBU, respectively. Additionally, in fish such as *Paracheirodon innesi*, exposure to DCF resulted in EC50-24h values of 120.6 mg/L for hyperventilation and LC50-24h values of 123.3 mg/L for mortality; for IBU, these effects were observed at EC50-24h values of 163.6 mg/L and LC50-24h values of 168.9 mg/L, respectively [4]. Previous studies have identified DCF and acetylsalicylic acid (ASA) as particularly harmful xenobiotics to aquatic life [5]. In addition, environmental risk assessments conducted in Mexico have found that compounds such as naproxen (NPX), DCF, 17β-estradiol (E2), and caffeine (CAF) have risk quotients (RQ) exceeding 1, indicating potential threats to regional ecosystems [6].

Globally, an assessment of these pollutants reveals a marked disparity in monitoring efforts and scientific output. According to the United Nations regional groups, Western Europe accounts for 77.5% of global research, while Latin America and the Caribbean (GRULAC) contribute just 1.6%. This imbalance also extends to the environmental matrices analyzed: 47% of studies focus on surface water and 40% on wastewater treatment plant effluents, leaving sediments with only 2% representation in the international literature [7].

In this context and given the growing evidence of emerging contaminants in Mexican aquatic ecosystems, it is necessary to understand not only their occurrence but also their distribution across environmental matrices and the processes governing their persistence.

Therefore, the objective of this study is to analyze the environmental fate of emerging contaminants reported in Mexico during the 2004–2025 period by compiling and systematizing studies conducted in freshwater bodies, groundwater, sediments, and wastewater treatment plants. Furthermore, the study identifies the most common contaminants, the environmental matrices in which they predominate, the processes associated with their transport and accumulation, and the available evidence on removal efficiency in conventional treatment systems, with the aim of providing a comprehensive overview of the magnitude and behavior of this environmental problem in the country.

## 2. Legal and Regulatory Framework Governing Water Quality in Mexico

The Official Mexican Standard NOM-001-SEMARNAT-2021 establishes the maximum permissible limits for parameters that treated wastewater must meet prior to its discharge into national water bodies, including soil. The regulated parameters include temperature, fats and oils, total suspended solids (TSS), chemical oxygen demand (COD), total organic carbon (TOC), total nitrogen (TN), total phosphorus (TP), pH, true color, metals such as As, Cd, Cr, Cu, Hg, Ni, and Pb, cyanides, helminth eggs, *Escherichia coli*, and fecal enterococci [8]. This update replaced NOM-001-SEMARNAT-1996 and no longer includes BOD_5_ as a primary control parameter [9].

For groundwater, Mexican regulations depend on the use or associated activity. When groundwater is intended for human consumption, it must comply with NOM-127-SSA1-2021, which establishes permissible limits for drinking water quality and corresponding water treatment processes, considering microbiological, physical, organoleptic, chemical, and radioactive parameters [10]. For activities involving the injection of water into the subsurface, NOM-014-CONAGUA-2003 establishes the requirements for the artificial recharge of aquifers with treated wastewater [11], while NOM-015-CONAGUA-2007 regulates the artificial infiltration of water into aquifers [12]. These standards aim to protect groundwater quality from induced recharge or infiltration processes; however, they do not include specific permissible limits or systematic monitoring programs for most emerging contaminants, such as PhACs, endocrine-disrupting compounds, personal care products, plastic additives, illicit drugs, metabolites, or transformation products.

In the United States, the Environmental Protection Agency reported and updated information on the Final PFAS National Primary Drinking Water Regulation on 18 May 2026. This regulation, originally announced in 2024, established legally enforceable Maximum Contaminant Levels for selected PFAS in drinking water, including 4 ng/L for perfluorooctanoic acid (PFOA) and perfluorooctanesulfonic acid (PFOS), 10 ng/L for perfluorohexanesulfonic acid (PFHxS), perfluorononanoic acid (PFNA), and hexafluoropropylene oxide dimer acid (HFPO-DA), as well as a hazard index for mixtures containing PFHxS, PFNA, HFPO-DA, and perfluorobutanesulfonic acid (PFBS). In 2026, EPA also announced proposed regulatory actions related to implementation and possible revision of some PFAS drinking-water requirements (U.S. Environmental Protection Agency, Washington, DC, USA, 2026) [13]. On the other hand, in the European Union, Directive 2008/105/EC [14] establishes environmental quality standards for priority substances and other pollutants in inland surface waters, transitional waters, and coastal waters. Among the compounds included are pollutants detected or discussed in Mexican aquatic matrices, such as azithromycin (AZM), carbamazepine (CBZ), clarithromycin (CLA), erythromycin (ERY), estrone (E1), glyphosate (GLY), DCF, IBU, PFAS, and triclosan (TCS), with regulatory limits ranging from ng/L to µg/L, depending on the compound and the type of water body [14]. For groundwater, Directive 2006/118/EC [15] establishes criteria to protect it from pollution and deterioration, including reference values for pesticides and other contaminants, as well as the possibility of setting threshold values for substances that could compromise the good chemical status of the aquifer. It sets a limit of 2.5 μg/L for CBZ and 0.1 μg/L for sulfamethoxazole (SMX) [15].

In Mexico, the National Water Commission (CONAGUA), through the National Water Information System (SINA), reports water quality indicators for surface, coastal, and groundwater bodies. For surface waters, parameters such as COD, BOD_5_, water temperature, fecal coliforms, *Escherichia coli*, fecal enterococci, dissolved oxygen saturation percentage, and acute toxicity are considered [16]. Additionally, SINA classifies water bodies into lotic, lentic, coastal, and groundwater systems. Lotic systems correspond to water bodies with continuous flow, such as rivers and streams; lentic systems correspond to bodies of water with little movement, such as lakes, lagoons, dams, or reservoirs; coastal systems correspond to coastal bodies of water, such as coastal lagoons, estuaries, bays, and river mouths; and groundwater systems mainly include wells and aquifers.

According to SINA, the 2024 national water quality statistics by water body type reported that 39.68% of lentic systems, 51.49% of lotic systems, and 45.49% of groundwater systems have heavily polluted water, while 19.05% of lentic systems, 19.68% of lotic systems, 14.29% of coastal systems, and 9.01% of groundwater systems were classified as “yellow,” indicating moderate contamination. This classification is based on BOD, COD, TSS, and *E. coli* parameters for lentic and lotic systems, while that for coastal systems is based on TSS, fecal coliforms, and fecal enterococci; furthermore, the classification of groundwater is based on the criteria of alkalinity, conductivity, total dissolved solids (TDS), fluorides, hardness, and nitrate nitrogen [17].

Overall, this regulatory overview shows that Mexican water quality regulations remain focused primarily on conventional physicochemical, microbiological, and toxicological parameters. Although there are standards for wastewater discharges, water for human use and consumption, artificial recharge, and infiltration into aquifers, most of the emerging contaminants reported in Mexican aquatic systems—such as PhACs, endocrine disruptors, personal care products, plastic additives, illicit drugs, metabolites, and transformation products—do not have specific permissible limits or systematic monitoring in national water quality programs. In contrast, regulatory examples from the United States and the European Union show that some emerging contaminants, such as PFAS, antibiotics, anti-inflammatory drugs, anticonvulsants, herbicides, and antimicrobial compounds, already have reference values or regulatory limits in other regulatory frameworks. This difference highlights a significant regulatory gap in Mexico and justifies expanding environmental monitoring to include non-conventional compounds.

## 3. Methodology

### 3.1. Study Design

A scoping review was conducted in accordance with the PRISMA-ScR guidelines to map, systematize, and synthesize the available evidence on the presence, distribution, environmental fate, persistence, and removal of emerging contaminants in Mexican aquatic matrices [18]. The study covers a two-decade period (2004–2025), providing a historical perspective on the evolution of this environmental issue in the country. This scoping review was not prospectively registered; however, it was conducted following the PRISMA-ScR guidelines.

### 3.2. Information Sources and Search Strategies

To gather the data, the bibliographic databases Scopus and Web of Science (WoS) were consulted, selected for their high impact and rigorous indexing of environmental sciences and biotechnology.

The literature search was conducted over approximately six months, and the final update of the Scopus and Web of Science searches was performed before preparing the revised manuscript. Records published between 2004 and 2025 were considered.

The search was conducted using search terms in both English and Spanish to ensure that both local scientific output and internationally published research were captured. Boolean operators (AND, OR) were used to combine terms in the title, abstract, and keyword fields. The main search was structured as follows:

Spanish: (“contaminantes emergentes” OR “compuestos activos farmacéuticos”) AND (“México” OR “lagunas” OR “acuífero” OR “sedimentos” OR “PTAR” OR “ecosistemas acuáticos”).

English: (“emerging contaminants” OR “pharmaceutical active compounds”) AND (“Mexico” OR “lagoons” OR “aquifer” OR “sediments” OR “WWTP” OR “aquatic ecosystems”).

As a complementary strategy to minimize selection bias, the backward snowball was applied. This involved manually tracking down and consulting the original primary sources cited in the review articles identified in the initial search.

### 3.3. Eligibility Criteria

We included original articles, book chapters, theses, and academic papers that reported empirical data on the detection, occurrence, distribution, concentration, removal, or environmental fate of emerging contaminants and associated compounds in Mexican environmental matrices. The matrices considered were inland and coastal surface waters, groundwater, cenotes, wetlands, reservoirs, sediments, and influents and effluents from wastewater treatment plants.

We excluded literature reviews without primary data, duplicate documents, studies not conducted in Mexico, studies focused exclusively on laboratory toxicological assays without data on environmental occurrence, and studies focused solely on conventional, regulated, or persistent organic pollutants that did not address emerging pollutants or associated compounds.

### 3.4. Study Selection

The identified records were organized in the Mendeley database for bibliographic management and screening. Subsequently, duplicate records were removed, and an initial screening was conducted based on the title, abstract, and keywords. Potentially relevant documents were reviewed in full to verify compliance with the eligibility criteria. Finally, the included studies were classified by environmental matrix, type of aquatic system, group of contaminants, and the availability of information on concentration, persistence, accumulation, or removal.

The process of identifying, screening, assessing eligibility, and including studies was summarized using a PRISMA-ScR-based flowchart.

### 3.5. Data Extraction and Synthesis

The following information was extracted from each included study: author and year of publication, geographic location, environmental matrix, type of aquatic system, group of contaminants, detected compounds, concentration range, analytical method used, and, when available, evidence regarding removal, persistence, accumulation, or environmental fate.

Data were extracted from tables and text. Compounds reported as detected but not quantified were included qualitatively in the occurrence tables and indicated as “detected; no concentration reported.” These compounds were not used for quantitative concentration-range comparisons. Compounds included in the analytical design of the original studies but not detected in environmental samples were not included in the occurrence tables.

The content analysis was structured around four main categories: surface water, groundwater, sediments, and influents/effluents from wastewater treatment plants.

Freshwater systems: rivers, lagoons, cenotes, wetlands, and reservoirs.

Groundwater systems: aquifers, wells, springs, and cenotes.

WWTPs: focused on removal efficiency and effluent concentrations.

Sediments: analysis of the accumulation and persistence of compounds in the solid phase of water bodies.

This compartmentalization enabled identification of spatial variations and specific trends in contaminant behavior across affected environmental compartments. Additionally, the reported concentrations were standardized to the same unit of measurement: ng/L for water and ng/g for sediments.

**Figure 1:**
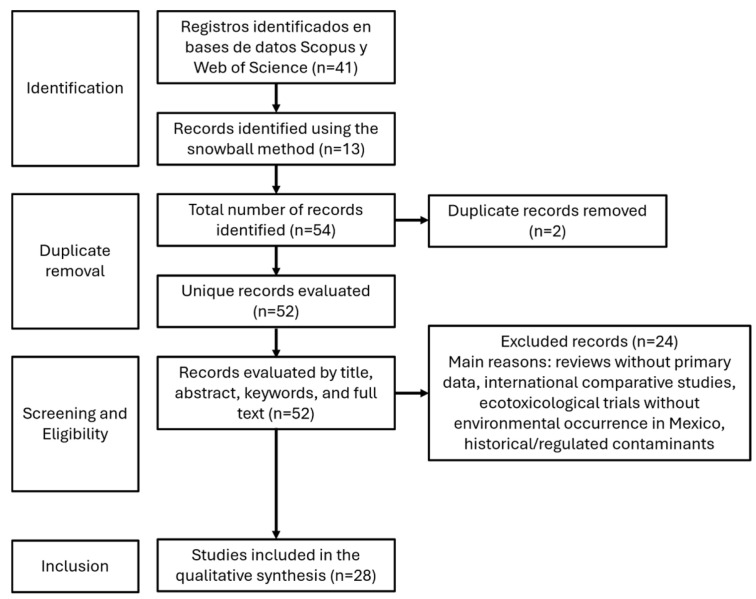
PRISMA-ScR flowchart of the process for identifying, screening, assessing eligibility, and including studies used in the scoping review.

A search of Scopus and Web of Science identified 41 records, and 13 additional documents were obtained through retrospective snowball sampling, for a total of 54 records. After removing two duplicates, 52 documents were evaluated based on their titles, abstracts, keywords, and full texts. Of these, 24 were excluded for not fully meeting the eligibility criteria, primarily because they were reviews lacking primary data, international comparative studies, ecotoxicological trials without environmental occurrence in Mexico, or studies focused on historical or regulated contaminants without a primary focus on emerging contaminants. Finally, 28 studies were included in the qualitative synthesis; Figure 1 shows the PRISMA-ScR diagram.

The included studies are documents containing empirical data on the occurrence, distribution, concentration, removal, accumulation, or environmental fate of emerging contaminants and associated compounds in Mexican aquatic matrices. In addition, a table was created to classify, by category, the emerging contaminants found—both qualitatively and quantitatively—in Mexican environmental matrices.

Government reports and official databases were also reviewed to contextualize the regulatory and water quality monitoring framework in Mexico. These sources included information from CONAGUA, SINA, and the Official Mexican Standards (NOMs) related to wastewater discharges, water quality for human consumption, artificial recharge, and infiltration into aquifers. However, these sources primarily report conventional water quality indicators, such as COD, BOD_5_, TSS, microbiological indicators, dissolved oxygen, acute toxicity, salinity-related parameters, and some inorganic constituents. Because EC concentrations are not systematically reported, they were used as contextual and regulatory sources but not as primary records of their occurrence for the quantitative synthesis of ECs in Mexican aquatic matrices.

## 4. Analytical Approaches Used to Detect Emerging Contaminants in Mexican Aquatic Matrices

EC analysis in Mexican aquatic matrices has been based primarily on processing samples collected in the field, followed by preparation, extraction, or preconcentration steps, and subsequently by instrumental or immunoenzymatic techniques for identification and quantification.

In karstic groundwater systems, where water flow can be rapid and variable, passive samplers are a suitable alternative for integrating exposure over extended periods. Metcalfe et al. used Semi-Permeable Membrane Devices (SPMDs) and Polar Organic Chemical Integrative Samplers (POCIS) in flooded caves in Tulum and Puerto Aventuras, which enabled the detection of PhACs, caffeine (CAF), cotinine (COT), cocaine (COC), benzoylecgonine (BE), TCS, nonylphenol, polycyclic aromatic hydrocarbons (PAHs), and chlorophenoxy herbicides [19]. This approach is useful in dynamic coastal aquifers, as it allows for the identification of potential sources of pollution from urban areas, tourism, and the maintenance of green spaces.

In surface waters, the studies reviewed have involved sampling in amber vials during one to three campaigns, with samples transported and stored at 4 °C until analysis. Because ECs are typically found in trace concentrations, generally in the range of ng/L to µg/L, most studies include preliminary extraction and preconcentration steps. Among the most commonly used strategies is solid-phase extraction (SPE), which is applied prior to instrumental analysis to improve analytical sensitivity and reduce matrix interference.

In surface waters, SPE has been routinely used as an extraction and preconcentration strategy, followed by gas chromatography coupled with mass spectrometry (GC-MS), for the determination of NPX, IBU, EDCs, ketoprofen (KTP), 4-nonylphenol (4-NP), bisphenol A (BPA), and the hormones E1, E2, 17α-ethinylestradiol (EE2), CAF, paraxanthine (PX), TCS, pentachlorophenol (PCP), and butylbenzyl phthalate (BBP) [20,21,22,23,24]. Furthermore, liquid–liquid extraction and SPE have also been previously used, prior to liquid chromatography techniques coupled with mass spectrometry, for the determination of PhACs, particularly NSAIDs [25,26]. In the case of GLY, water sample preparation involved filtration through 0.4 μm cellulose cartridges or membranes, followed by quantification by ELISA [27]. Although these methods are useful for initial assessments and field monitoring, supplementing them with chromatographic techniques is important for confirming results and expanding metabolite and transformation product detection.

For EC analysis in sediments, the reviewed studies have considered surface sediments ranging from 0–5 cm to as deep as 10 cm, 25 cm sediment cores, as well as sediments collected using sediment traps. The number of sampling points has varied, with studies including between 5 and 18 sites, depending on the matrix, the environmental system, and the monitoring objective. In these matrices, sample preparation is typically more labor-intensive than in water, due to the interaction of contaminants with organic matter, fine particles, and mineral phases. Processes such as drying, freeze-drying, grinding, sieving, solvent extraction, cleaning, and preconcentration have been reported in the literature prior to instrumental analysis.

In sediments, Ronderos-Lara et al. (2022) used ultrasound-assisted extraction followed by SPE to determine IBU, NPX, 4-NP, BPA, E2, and EE2 by GC-MS [24]. In the Veracruz Reef System, Salazar-Remigio et al. (2025) analyzed surface and trap sediments using ultrasound-assisted extraction with methanol for bisphenols (BPs) and with dichloromethane for phthalic acid esters (PAEs); subsequently, the extracts were purified using SPE and quantified by GC-MS/MS [28]. Additionally, in sediments from Laguna de La Paz, Becerra-Rueda et al. (2024) [29] reported using accelerated solvent extraction (also known as pressurized liquid extraction) with methanol, followed by purification via SPE and quantification of CBZ, CAF, SMX, and ciprofloxacin (CIP) by HPLC-MS/MS.

Single and composite samples have been collected from wastewater inflows and effluents during both the dry and rainy seasons. Among the preparation, extraction, and preconcentration strategies used were solid-phase microextraction (SPME), liquid–liquid extraction, and extraction into acidic and basic fractions, followed by SPE. Peña-Álvarez and Castillo-Alanís (2015) [30] used SPME for simultaneous extraction and preconcentration, followed by GC-MS, to determine IBU, NPX, TCS, BPA, and chlorophenol (CP) in influent and effluent samples from wastewater treatment plants. In contrast, Estrada-Arriaga et al. (2016) applied a multi-residue analytical workflow based on acid and base extractions, cleanup via SPE using Oasis Hydrophilic–Lipophilic Balance (HLB) cartridges, and determination by HPLC-MS/MS to assess 55 ECs in influents and effluents from full-scale WWTPs in Guanajuato and Valle de Bravo [30,31].

Similarly, Robledo Zacarías et al. (2017) [32] used liquid–liquid extraction at different pH values on influent and effluent samples from the Atapaneo WWTP in Morelia, followed by Fourier-transform infrared spectroscopy (FTIR) as a preliminary technique and electrospray ionization-time-of-flight mass spectrometry (ESI-MS-TOF) for the identification and quantification of PhACs, drugs of abuse, hormones, and polyethylene glycol. In this case, FTIR should be considered a preliminary or screening tool, while ESI-MS-TOF offers greater capability for identifying compounds in complex mixtures [32].

In a hospital effluent from Toluca, 11 PhACs were quantified using UHPLC, including GLB, MTF, ATE, MTP, PEN G, PEN V, E2, DCF, IBU, NPX, and PCM, highlighting the importance of hospitals as point sources of pollution [33]. This type of analytical approach is relevant because it allows for the detection of polar, thermally unstable, or low-volatility compounds that are not suitable for direct analysis by GC-MS.

Overall, Mexican studies show that the choice of analytical approach depends on both the environmental matrix and the chemical group being evaluated. GC-MS and GC-MS/MS have been used primarily for phthalates, bisphenols, alkylphenols, hormones, xanthines, and some industrial compounds; LC-MS/MS, HPLC-MS/MS, or UHPLC-MS/MS are more suitable for PhACs, antibiotics, polar compounds, and some metabolites; ELISA has been applied to the monitoring of herbicides such as glyphosate; and passive samplers are useful in karst systems or in environments with high hydrodynamic variability.

In general, the analytical approaches used in Mexican aquatic matrices have evolved from the specific detection of a limited number of compounds toward broader multiresidue analysis strategies. However, metabolites and transformation products remain understudied in Mexico. Therefore, it is necessary to incorporate high-resolution methodologies, suspect-based and non-targeted analyses, as well as multiresidue strategies, to expand the identification of chemical pollutants, assess their environmental fate, and more comprehensively estimate ecological and human health risks.

## 5. Classification of Emerging Contaminants and Associated Compounds

The classification was based on the pollutant categories reported in the studies included in this review. These groups represent the main categories detected in Mexican aquatic systems, including pharmaceutically active compounds, endocrine disruptors, personal care products, pesticides, industrial additives, illicit drugs, xanthines, phthalates, bisphenols, and perfluoroalkyl and polyfluoroalkyl substances. Their inclusion is environmentally relevant because these compounds are associated with various anthropogenic sources, such as urban wastewater, hospital effluents, industrial discharges, agricultural runoff, tourism activities, and the use and disposal of consumer products.

On the other hand, transformation products and metabolites have been considered separately. Although these compounds have been detected less frequently in Mexican aquatic systems than their parent compounds, their inclusion is important because some may be persistent in the environment, retain their biological activity, or serve as tracers of specific pollution sources.

Table 1 classifies pollutants, while Table 2 lists the metabolites of these pollutants detected in Mexican environmental matrices.

As shown in Table 2, the presence and quantification of metabolites of emerging contaminants remain poorly studied, representing a significant gap.

Overall, this classification shows that the emerging contaminants detected in Mexican aquatic matrices belong to chemically diverse groups, with different sources, environmental behaviors, and levels of persistence.

## 6. Emerging Contaminants in Environmental Matrices

### 6.1. Emerging Contaminants in Water Bodies

The pathways by which ECs enter environmental compartments are urban and industrial wastewater discharges, with or without prior treatment.

The Mexico City metropolitan area obtains water for human consumption from both groundwater and surface water sources; the local aquifer supplies approximately 66% of total demand, while the Lerma aquifer and the Cutzamala dam system contribute approximately 8% and 33%, respectively. Therefore, detecting emerging contaminants in these sources is of environmental importance because they are directly linked to water supply systems. Monitoring of seven wells and four reservoirs revealed the presence of DCF, TCS, BPA, 4-NP, NPX, IBU, KTP, salicylic acid (SA), di(2-ethylhexyl) phthalate (DEHP), benzyl butyl phthalate (BBP), and gemfibrozil (GFZ) in surface waters [34].

These compounds correspond to the groups summarized in Table 3, which include NSAIDs, plasticizers, personal care products, endocrine-disrupting compounds, and lipid regulators, indicating that EDC contamination is not limited to rivers affected by wastewater but also affects water bodies used for urban water supply.

In this context, Table 3 summarizes the emerging contaminants detected in inland and coastal surface waters throughout Mexico, enabling comparisons across regions, water-body types, and contaminant groups.

A study on the global concentration of estrogens in aquatic ecosystems concluded that Morocco has the highest levels of E1 and E2, while estriol (E3) is most prevalent in Australia and EE2 in Canada. As for pesticides such as dichlorodiphenyltrichloroethane (DDT), diazinon (DZN), and heptachlor (HEP), Bangladesh has the highest concentrations. On the other hand, Canada leads in BPA, and China and Japan have the highest levels of PFOS and PFOA, respectively. Finally, in the Netherlands and Argentina, the surfactants nonylphenol (NP) and octylphenol (OP) predominate, while the highest concentrations of PhACs such as DCF, NPX, and IBU were recorded in Pakistan, India, and Spain [37].

When comparing these data with the Mexican results summarized in Table 3, it can be seen that in Mexico, surface waters are also affected by PhACs, endocrine disruptors, industrial additives, pesticides, and xanthines; however, the highest concentrations are primarily associated with urbanized watersheds and systems that receive wastewater inflows. For example, the Apatlaco River watershed showed elevated concentrations of endocrine-disrupting compounds, such as 17α-ethinylestradiol, bisphenol A, 17β-estradiol, and 4-nonylphenol, while the Santa Catarina River had the highest concentrations of benzenesulfonamide, di(2-ethylhexyl) phthalate, and bisphenol A. These differences suggest that land use, wastewater discharges, industrial activity, population density, agricultural practices, and treatment capacity significantly influence the patterns of occurrence and concentration of emerging contaminants in Mexican aquatic systems.

**Figure 2:**
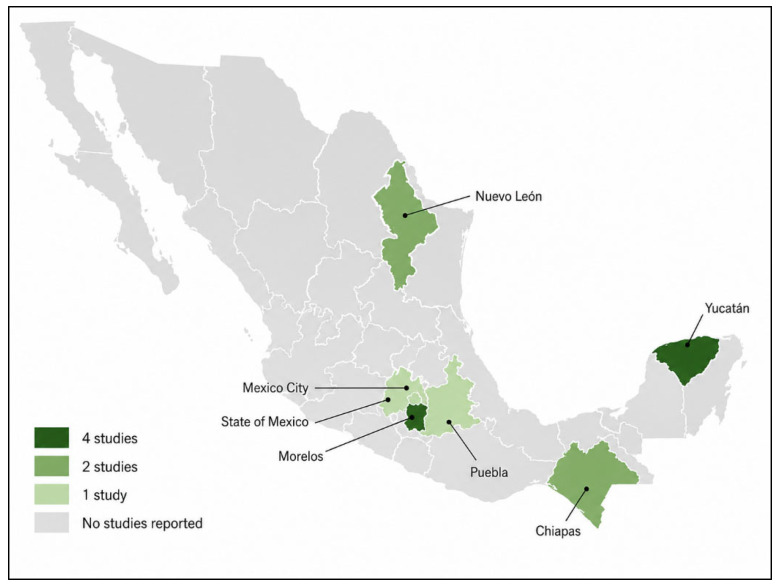
Geographical distribution of studies reporting emerging contaminants in Mexican water bodies.

Based on Table 3, research on ECs in Mexican aquatic ecosystems was conducted that research on the presence of ECs in Mexican aquatic ecosystems was conducted between 2013 and 2024; a significant proportion of these studies are concentrated in the state of Morelos. Both inland and coastal surface water systems have been studied. Figure 2 shows the spatial distribution of studies reporting the presence of emerging contaminants in Mexico’s freshwater systems.

The urban rivers Apatlaco, Cuautla, and Santa Catarina exhibit the greatest diversity of pollutants, attributable to urban influence.

The reported concentrations correspond to different groups of ECs, notably PhACs, primarily NSAIDs such as ibuprofen, naproxen, ketoprofen, and diclofenac, as well as acetaminophen, the antibiotics trimethoprim and sulfamethoxazole, the antihypertensive atenolol, the lipid-regulating agents bezafibrate and gemfibrozil, and carbamazepine. Also reported are EDCs such as estrone, estradiol, ethinylestradiol, bisphenol A, 4-nonylphenol, and 4-t-octylphenol; personal care products such as triclosan; plasticizers such as benzyl butyl phthalate and di(2-ethylhexyl) phthalate; the pesticide glyphosate; and markers of urban pollution such as caffeine and paraxanthine. These compounds are primarily associated with urban wastewater discharges, consumer products, and, in the case of glyphosate, agricultural activities.

The highest concentrations were reported in the Santa Catarina River in Nuevo León, where the compound with the highest concentration was benzene sulfonamide, followed by di(2-ethylhexyl) phthalate and bisphenol A, possibly associated with urban discharges, runoff, and industrial activity in the Monterrey metropolitan area. Meanwhile, in the Apatlaco River basin in Morelos, high concentrations of ethinyl estradiol, bisphenol A, estradiol, and 4-nonylphenol were reported, suggesting a significant influence of urban activity, population density, and wastewater discharges.

The presence of CAF in aqueous matrices is explained by its low log Kow (<1), which promotes its persistence in the dissolved phase and its transport in water bodies affected by domestic discharges [38].

A recent study evaluated the bioaccumulation of ECs in wild fish caught in two coastal lagoons located on the Pacific Ocean: Barra de Navidad Lagoon in Jalisco and Cuyutlán Lagoon in Colima. The results showed the presence of the following NSAIDs in all species analyzed: DCF, IBU, ketorolac (KET), and NPX. The highest contaminant concentration in fish from Barra de Navidad Lagoon was IBU, at 0.01–0.31 µg/kg, whereas in Cuyutlán Lagoon it was NPX, at 0.01–0.28 µg/kg. Additionally, the highest accumulation was detected in carnivorous species (*Caranx caninus*, *Synodus lacertinus*) and detritivorous species (*Ariopsis felis*, *Mugil curema*), suggesting that NSAIDs are present in both the water column and the sediments [39].

In general, Mexico’s surface waters are the most studied aquatic ecosystem and exhibit the greatest diversity of emerging contaminants. Urban rivers, such as the Apatlaco, Cuautla, and Santa Catarina, contain a wide range of PhACs, endocrine-disrupting compounds, xanthines, industrial additives, personal care products, and pesticides, indicating that wastewater—whether untreated or inadequately treated—remains the primary cause of pollution. These findings show that surface waters act as direct recipients of multiple anthropogenic inputs and should remain a priority for national monitoring programs.

### 6.2. Emerging Contaminants in Groundwater

The underground geological formations that store and allow the flow of water are called aquifers, which are classified as unconfined, confined, or karst.

In general, contamination of groundwater stored or transported in aquifers results from human activities and aquifer hydrogeological characteristics. Factors influencing vulnerability include soil depth, topography, precipitation, recharge conditions, groundwater flow, and pollutant loads from point or nonpoint sources, such as landfills, agricultural, livestock, urban, and industrial activities [40].

Urban, industrial, and stormwater runoff in Mexico City is collected through the general drainage system and channeled to the Tula Valley in Hidalgo, where it has been used untreated for agricultural irrigation since 1912. As a result of this practice, analysis of four agricultural plots revealed the presence of emerging contaminants, including IBU, NPX, CBZ, TCS, BPA, 4-NP, dibutyl phthalate (DBP), BBP, and DEHP, at concentrations above the limit of detection. The highest concentrations were observed in the plots closest to the irrigation canal, while only TCS and BPA were detected in the aquifer water [41]. A complementary study in the same area evaluated the sorption and desorption of NPX, CBZ, and TCS in agricultural soils irrigated with raw wastewater. To this end, soil samples were collected from two soil profile intervals: 0–10 cm (plow layer) and 30–40 cm (subsurface layer). The initial concentrations in the surface soil were 2.7, 6.2, and 9.7 ng/g for NPX, CBZ, and TCS, respectively, while at 30–40 cm they were 0.7, 1.4, and 6.3 ng/g, respectively. These results demonstrate the accumulation of emerging contaminants in both the surface and subsurface soil layers, associated with prolonged wastewater irrigation. Similarly, the wastewater used in the sorption/desorption tests contained 6358 ng/L of NPX, 730 ng/L of CBZ, and 1401 ng/L of TCS. The results showed that TCS had the highest retention in the soil, due to its hydrophobicity and affinity for organic matter, followed by CBZ, a less hydrophobic compound but one capable of interacting with organic matter and clays. In contrast, NPX exhibited the lowest sorption capacity, attributable to its negative charge and soil pH, which promote repulsion from soil particles. Furthermore, desorption tests demonstrated that NPX and CBZ can be re-released from the soil, implying a potential risk of mobilization into aquifers during rainfall events or changes in irrigation water composition. In contrast, TCS exhibited virtually irreversible sorption and therefore tends to remain retained in the soil [42].

In addition, the local aquifer in the Mexico City metropolitan area and the Lerma aquifer are contaminated with SA, DCF, DEHP, BBP, TCS, BPA, and 4-NP at lower concentrations, indicating that TCS and BPA are contaminants that infiltrate the aquifers, in addition to SA, DCF, and the endocrine disruptors DEHP, BBP, and 4-NP [34].

Groundwater from the Riviera Maya’s coastal karst system flows toward the coastal areas of the Mexican Caribbean and thus can act as a pathway for the transport of contaminants from urban and tourist areas to marine ecosystems. Metcalfe et al. (2011) [19] used passive samplers in flooded caves beneath Tulum and in Puerto Aventuras for approximately one month. In addition to PhACs, they detected CAF, COT, COC, and BE, as well as TCS, nonylphenol, PAHs, and chlorophenoxy herbicides. PhACs, stimulants, illicit drugs, and personal care products were attributed primarily to treated or untreated domestic wastewater, while the chlorophenoxy herbicides detected in Puerto Aventuras were linked to the maintenance of green spaces and a nearby golf course. These results highlight the vulnerability of the coastal karst aquifer to pollution resulting from urban and tourism development [19].

Agricultural communities are at risk of pesticide leaching into groundwater from currently used pesticides. The municipality of Hopelchén, Campeche, is a region that produces soybeans, corn, sorghum, and tomatoes, where the broad-spectrum herbicide GLY is used. Human exposure can occur directly, among farmers who apply herbicides, or indirectly, through the consumption of contaminated water and food. Because GLY can be excreted unchanged in urine, this matrix has been used as an indicator of recent exposure. Among subsistence farmers in Francisco J. Mújica, a GLY concentration of 470 ng/L was reported in human urine, while the urban reference group in Campeche had a concentration of 220 ng/L, attributable to direct and indirect exposures, respectively. Bottled water in the region is produced from groundwater treated by reverse osmosis. However, bottled water sold in Mérida, Yucatán, contained 350 ng/L of GLY, while that produced in the town of Ich-Ek reached 650 ng/L [43]. In the community of Tenampulco, Puebla, GLY was also detected in surface and groundwater; a survey revealed that farmers use GLY, 72% of whom mix it with 2,4-D and paraquat; 56% do not use adequate personal protective equipment; in addition to improper handling of containers, 50% burn them and 22% dispose of them with regular trash due to the lack of pesticide container collection programs [27]. These results are linked to the presence of GLY in the area’s surface and groundwater, due to agricultural activity and poor pesticide application and container disposal practices, highlighting the need to improve water purification processes for human consumption and to assess the quality of the source water.

In karst regions, cenotes are natural openings connected to the aquifer through networks of porous rock, cracks, and fractures, and thus function as surface manifestations of groundwater. An assessment conducted at 10 cenotes and 1 submarine groundwater discharge site in Quintana Roo, between Playa del Carmen and Tulum, reported elevated concentrations of lead, nitrates, and *Escherichia coli*. In addition, 34 antibiotic-resistant *E. coli* strains were identified in nine cenotes, along with PFAS, primarily PFOS and PFHxA, with total concentrations ranging from 0.68 to 10.71 ng/L. This highlights the need to improve water purification processes for human consumption and to assess the quality of the source water [44].

**Figure 3:**
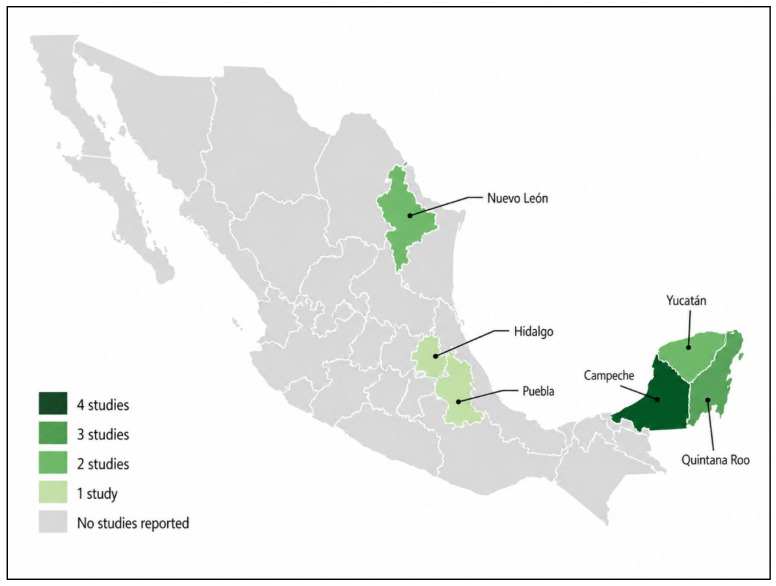
Geographical distribution of studies reporting emerging contaminants in Mexican groundwater.

Table 4 shows that between 2007 and 2024, only a few groundwater sites in Mexico have been studied. These sites exhibit contamination by PhACs, EDCs, personal care products, phthalates, currently used pesticides, illicit drugs, and industrial compounds [19,27,36,43,44,45,46]. Figure 3 shows the spatial distribution of studies reporting the presence of emerging contaminants in groundwater.

The diversity of ECs present in the Cerro Colorado spring is attributed to possible artificial recharge with wastewater from the Emisor Central in Mexico. Gibson et al. assessed the concentration of ECs in both the wastewater from the Emisor Central and the groundwater at the Cerro Colorado spring; their results showed nearly the same contaminants, but at lower concentrations, attributable to infiltration and soil attenuation; however, 2,4-dichlorophenoxyacetic acid, gemfibrozil, ketoprofen, and diclofenac were not detected in the groundwater [45].

The ECs with the highest concentrations in the groundwater associated with the Santa Catarina River are benzenesulfonamide, di(2-ethylhexyl) phthalate, and bisphenol A, suggesting that this groundwater receives inflows from urban and industrial sources. These compounds were determined using SPE-GC-MS [36], an analytical method suitable for industrial compounds such as bisphenols and phthalates. The BSA concentrations recorded in groundwater were particularly high, reaching levels on the order of mg/L, suggesting that this compound may be associated with localized or diffuse inputs rather than background contamination. Since Santa Catarina is located within the Monterrey metropolitan area, a highly urbanized and industrialized region, possible sources include industrial discharges, urban runoff, leaks from wastewater infrastructure, leachates from plastic waste, and inputs associated with the use or degradation of industrial additives. However, the study that identified these contaminants did not include a source tracking analysis. Therefore, elevated levels of BSA highlight the need for targeted monitoring of industrial organic compounds in urban aquifers and additional studies to identify their sources.

In the karst areas of the Yucatán Peninsula, where the Kikil and Chac Mool cenotes are also located, there is greater vulnerability due to the rapid movement of water through cracks and channels, which reduces the soil’s natural filtration capacity. In the cenotes, only the antibiotic tetracycline was detected; however, its concentration was not quantified. On the other hand, in the Riviera Maya, the influence of urban and tourism-related pollution is evident. Specifically, in Tulum, triclosan was detected and attributed to personal care products, along with cocaine and its metabolite benzoylecgonine, which were associated with urban and tourism wastewater. In Puerto Aventuras, NSAIDs such as ibuprofen and naproxen, as well as caffeine, were attributed to urban pollution, while 2,4-dichlorophenoxyacetic acid was linked to pesticide seepage [19].

Hopelchén and Tenampulco show the presence of glyphosate in surface and groundwater due to infiltration, at concentrations ranging from hundreds to thousands of ng/L, highlighting the need to assess ecological risk to biota [27,43].

On the other hand, in Santa Catarina, extreme concentrations of benzenesulfonamide, followed by di(2-ethylhexyl) phthalate and bisphenol A, suggest an urban and industrial influence associated with plastic waste [36].

In general, studies on groundwater indicate that emerging contaminants can infiltrate aquifers through wastewater discharges, agricultural activities, tourism-related discharges, septic systems, and urban development. This is particularly relevant in karst systems, where rapid water flow and high connectivity between surface and subsurface compartments can facilitate the transport of contaminants. Therefore, groundwater monitoring should be strengthened, especially in vulnerable aquifers that supply drinking water or are located in highly urbanized, agricultural, or tourist regions.

### 6.3. Emerging Contaminants in Sediments

Sediments in aquatic ecosystems play an important role in the environmental fate of pollutants, as these pollutants can remain dissolved in the water column or be adsorbed onto suspended and sedimentary particles, depending on their physicochemical properties. The adsorption of pollutants onto sediments facilitates their transfer from the aqueous phase to the solid phase, allowing for their accumulation and persistence in the environment. As a result, sediments can act as both sinks and secondary sources of pollution. In response to changes in environmental conditions such as variations in pH, dissolved oxygen, salinity, or sediment resuspension, retained pollutants may be released back into the water column [47].

The coastal lagoons of the Gulf of Mexico are of great ecological and economic importance; however, they have been affected by eutrophication from urban wastewater, agricultural runoff, and discharges associated with various human activities. In this context, the sediments of Mandinga Lagoon in Veracruz, Términos Lagoon in Campeche, and Chetumal Bay in Quintana Roo are contaminated with PAHs. Similarly, sediments in lagoon systems such as Tampamachoco in Alvarado and El Yucateco and Sabancuy in Campeche contain organochlorine pesticides [48,49,50]. In addition, an assessment of sediments in the Alvarado lagoon system in Veracruz, which consists of more than 100 coastal brackish lagoons and seasonally flooded areas, revealed the presence of hexachlorocyclohexanes, cyclodiene, methoxychlor, and heptachlor [51].

On the other hand, in the Veracruz Reef System (SAV), an area designated as a protected natural area, the presence of organochlorine pesticides, as well as bisphenols and phthalic acid esters, has been reported in surface and trap sediments, with BPA (0.66 ng/g) and DBP (2.58 × 10^3^ ng/g) being the most prevalent [28,52].

These findings indicate that sediments act as reservoirs for persistent substances and underscore their importance as a key environmental matrix for assessing historical contamination and the associated environmental risks. It should be noted that a synergistic effect has been observed in the toxicity of contaminant mixtures; therefore, it is necessary to assess the ecotoxicological risk associated with real-world mixtures.

On the other hand, La Paz Lagoon in Baja California Sur is biogeochemically connected to the Gulf of California and characterized by hypersaline waters and reverse estuarine circulation. This system receives wastewater discharges year-round, either untreated or partially treated in maturation ponds, as reflected by the presence of pharmaceutical residues in the sediments. CAF, a highly water-soluble substance, was detected in this system; its presence in sediments suggests recent or ongoing inputs of inadequately treated wastewater. In addition, CBZ, CIP, and SMX were detected, with SMX having the highest concentrations and being the only compound posing a high environmental risk to benthic organisms [29].

Table 5 presents the results of research on emerging contaminants in sediments from aquatic ecosystems.

Generally, research on contaminants in sediments focuses on the surface layer, that is, the top 5 cm. For this reason, the study by Becerra-Rueda et al. (2024) [29] stands out, as it not only analyzed surface sediments but also evaluated a 25 cm deep sediment core, which allowed them to observe the vertical distribution of contaminants. Assuming a sedimentation rate of 6.5 mm/year, the authors estimated that the core represents approximately 46 years of sediment deposition. In this core, caffeine and sulfamethoxazole were detected throughout the sediment column, with higher concentrations in the top 6 cm; carbamazepine was quantified primarily up to 15 cm, while ciprofloxacin was detectable down to 21 cm. This distribution suggests that the retention and mobility of PhACs in sediments depend on their physicochemical properties, total organic carbon content, and hydrodynamic conditions in the lagoon [29].

**Figure 4:**
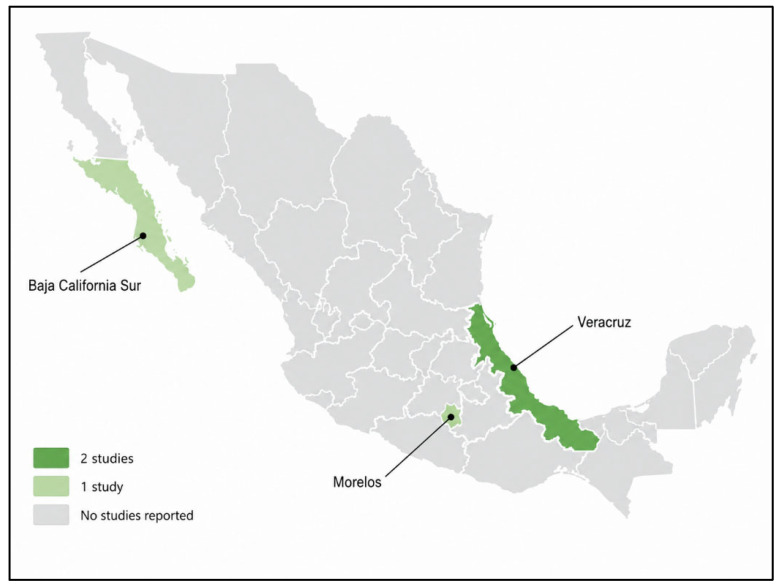
Geographical distribution of studies reporting emerging contaminants in Mexican sediments.

Figure 4 shows the spatial distribution of studies reporting the presence of emerging contaminants in Mexican sediments. Based on this figure and Table 5, there is a clear need for further research into emerging contaminants present in the sediments of aquatic ecosystems.

PhACs and EDCs were detected in sediments from the Apatlaco River and La Paz Lagoon, while BPs and PAEs were reported in the SAV. In the urban system analyzed, NSAIDs and hormones predominate, while PAEs predominate in the reef system.

In the sediments of the Apatlaco River, concentrations of PhACs, EDCs, and hormones are in the order of ng/g and are attributed primarily to urban wastewater discharges. E2 and EE2 have also been detected in sediments from the Venice Lagoon in Italy [53].

On the other hand, phthalates are present at levels in the order of thousands of ng/g, suggesting an affinity for sedimentary organic matter; their presence is attributed to plastic waste, urban effluents, port activity, and continental inputs transported by rivers. Analysis of trap sediments showed that PAEs continue to be actively deposited on the seafloor, confirming that these sediments act as sinks for phthalic acid esters. This finding, combined with bisphenol concentrations, indicates the need to strengthen environmental monitoring and implement remediation measures in protected natural areas such as the SAV.

In general, studies on sediments in Mexico remain scarce, but they show that sediments act as important sinks for emerging contaminants, particularly hydrophobic and particle-bound compounds such as phthalates, bisphenols, alkylphenols, hormones, and some PhACs. The detection of these compounds in urban rivers, coastal lagoons, and reef systems indicates that sediments can integrate long-term pollution signals and may also act as secondary sources upon resuspension or changes in physicochemical conditions. Therefore, sediments should be incorporated more systematically into monitoring programs, especially in protected coastal ecosystems and in areas receiving urban, industrial, or port discharges.

### 6.4. Emerging Contaminants in Effluents and Wastewater Treatment Plants

In Mexico, two issues related to wastewater treatment plants (WWTPs) are insufficient installed capacity and the inefficiency of conventional treatment methods in addressing ECs. Table 6 summarizes the ECs found in Mexican WWTPs.

In the absence of wastewater treatment plants, or when they are insufficient, wastewater is discharged into coastal lagoons or oxidation ponds. Another common practice in Mexico is the use of wastewater for agricultural irrigation.

The Mexico City Metropolitan Area combines wastewater from domestic and industrial sources, stormwater, and runoff and discharges it through three drainage channels: the Central Outfall, the Western Interceptor, and the Grand Canal, with an approximate flow rate of 50 m^3^/s, without prior treatment for use in agricultural irrigation. The concentration of ECs in the raw wastewater from the Emisor Central drainage channel was evaluated. The results showed concentrations of 4380–5090 ng/L of IBU, 15,220–16,650 ng/L of NPX, 1720–6360 ng/L of DCF, 11,000–22,400 ng/L of 4-NP, 660–2040 ng/L TCS, 770–2500 ng/L BPA, 710–1240 ng/L BBP, 356,000–702,000 ng/L DEHP, 44–80 ng/L E1, and 18–22 ng/L E2 [45].

It is worth noting that DEHP and 4-NP had the highest concentrations; furthermore, both are potential endocrine disruptors. This flow is responsible for the artificial recharge of the aquifer from which the Cerro Colorado spring in Tula, Hidalgo, emerges [45]. The use of untreated wastewater for agricultural irrigation carries the risk of introducing the ECs it contains into the food chain.

Consumption trends for PhACs are unknown. However, the hormones E1, E2, and EE2 have been quantified at two wastewater sampling sites in Mexico City. At the Central Emitter site, the endocrine disruptors E1, E2, and EE2 had concentrations of 14–54 ng/L, 12–27 ng/L, and >8 ng/L, respectively, while in the Grand Canal, concentrations of 4–93 ng/L were reported for these compounds [54].

A study of hospital effluent from Clinic 221 of the Mexican Social Security Institute (IMSS) in Toluca, State of Mexico, reported that this effluent is discharged untreated into the municipal sewer system and subsequently reaches a wastewater treatment plant (WWTP) with activated sludge and aerobic treatments [33].

At this point source of contamination, 11 PhACs were detected: 1,920,000 ± 20,000 ng/L of glibenclamide (GLB), 1,310,000 ± 20,000 ng/L of metformin (MTF), 200,000 ± 100 ng/L of ATE, 2,020,000 ± 300,000 ng/L of metoprolol (MTP), 3,770,000 ± 30,000 ng/L of penicillin G (PEN G), 420,000 ± 10,000 ng/L of penicillin V (PEN V), 80,000 ± 1000 ng/L of E2, 590,000 ± 300,000 ng/L of DCF, 620,000 ± 400,000 ng/L of IBU, 1,790,000 ± 200,000 ng/L of NPX, and 2,660,000 ± 20,000 ng/L of PCM [33]. These high concentrations are attributed to the hospital origin of the effluent and the absence of pretreatment. It should be noted that hospital effluents can act as significant point sources of pharmaceutically active compounds, antibiotics, anti-inflammatory drugs, hormones, and other contaminants; therefore, they must be taken into account in specific strategies for control, treatment, and environmental monitoring.

In addition, an assessment of five wastewater effluents in the coastal area of Cihuatlán, Jalisco, detected PCP, DCF, IBU, E2, and KET. Based on ecotoxicological toxicity equivalents, the ECs PCP, DCF, IBU, and KET were classified as toxic, while E2 was classified as highly toxic according to the Veith and Konasewick index [55].

**Figure 5:**
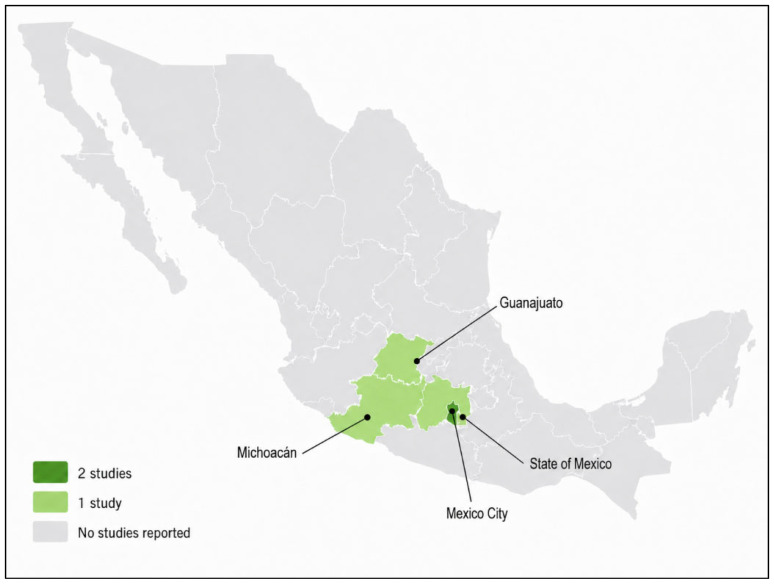
Geographical distribution of studies reporting emerging contaminants in Mexican WWTPs.

Figure 5 shows the spatial distribution of studies reporting the presence of emerging contaminants in Mexican wastewater treatment plants.

Despite limited research on influents and effluents from Mexican wastewater treatment plants (WWTPs) conducted between 2015 and 2017, it is evident that various groups of ECs, including PhACs, personal care products, endocrine disruptors, industrial compounds, and urban metabolites, are received and discharged. Although effluent concentrations are lower than those in the influent, they remain significant, indicating that these substances are not completely removed and are discharged into receiving waters.

In the influents, the pharmaceuticals with the highest concentrations were naproxen, metformin, acetaminophen, caffeine, and ibuprofen, as well as the metabolites paraxanthine and theophylline, whose presence is attributed primarily to urban discharges and human consumption patterns. Naproxen reached concentrations of up to 54,000 ng/L in the influents at the Ciudad Universitaria, Coyoacán, and Cerro de la Estrella WWTPs, while at the Guanajuato WWTP, maximum concentrations of 94,600 ng/L were reported for the evaluated ECs.

On the other hand, the significant concentrations of polyethylene glycol, tetracycline, amoxicillin, and lormetazepam in the effluent from the Atapaneo WWTP suggest that this plant may act as a point source of pollution for the receiving water body. Polyethylene glycol stands out, at 135,000 ng/L, attributable to industrial or urban-industrial discharges, followed by tetracycline, amoxicillin, and lormetazepam, associated with the use of antibiotics and psychotropic drugs.

In general, the available data show that conventional wastewater treatment plants in Mexico reduce the concentration of some emerging contaminants, but do not guarantee their complete removal. Compounds such as caffeine, acetaminophen, and ibuprofen can be effectively reduced through biological treatment, while carbamazepine, diclofenac, sulfamethoxazole, metoprolol, various antibiotics, psychotropic PhACs, and their metabolites tend to persist. Consequently, treated effluents may continue to act as point sources of pollution for rivers, reservoirs, agricultural soils, and coastal systems, highlighting the need for multi-contaminant monitoring and the evaluation of advanced or combined treatment processes.

## 7. Efficiency of Emerging Contaminant Removal at Wastewater Treatment Plants

The removal of contaminants in wastewater treatment plants depends on the configuration of the treatment train and the physicochemical properties of the compounds. In general, primary treatments are aimed at removing suspended solids and particulate matter, while secondary treatments—such as biological processes involving aeration, sedimentation, and activated sludge—facilitate the removal of biodegradable organic matter and nutrients [56]. Tertiary or polishing treatments, such as chlorination, ozonation, ultraviolet radiation, Fenton processes, activated carbon adsorption, or combined systems, can contribute to disinfection and the further removal of persistent contaminants.

Table 7 summarizes the treatment processes used in various WWTPs and the EC removal efficiencies reported in the reviewed studies.

Estrada-Arriaga et al. compared the presence and removal of ECs at two Mexican wastewater treatment plants: one in Guanajuato and the other in Valle de Bravo. During the rainy season, they identified 55 and 41 ECs, respectively, while during the dry season they detected 53 and 44 ECs. Concentrations ranged from 0.69 to 94,600 ng/L, with higher values during the dry season, attributable to lower dilution; in contrast, during the rainy season, concentrations decreased due to increased flow. The compounds with the highest concentrations were MTF, PCM, CAF, PX, THEO, NPX, ADT, SMX, CIP, and valsartan [31].

The Guanajuato WWTP, which uses oxidation ditches with simultaneous nitrification-denitrification and UV disinfection, achieved an overall EC removal rate of only 20–22%. In contrast, the Valle de Bravo WWTP, which operates with anaerobic/anoxic/aerobic reactors and combined disinfection using ClO_2_ and UV light, achieved overall removal rates of 66–80%. This difference demonstrates that the configuration of the biological system, hydraulic retention time, solids retention time, and the combination of disinfection processes influence EC removal.

Despite these differences, persistent compounds were identified at both WWTPs. CBZ, SMX, MTP, CLA, ERY-H_2_O, ofloxacin, norfloxacin, diazepam, alprazolam, fluoxetine, sertraline, propranolol, meprobamate, hydrochlorothiazide, diphenhydramine, and albuterol showed removal rates below 50%. This is significant because effluents from these WWTPs are discharged into the Guanajuato and Amanalco rivers, thereby serving as point sources of chemical pollution to the receiving water bodies [31].

In addition, Estrada-Arriaga et al. evaluated three physicochemical post-treatment processes applied to secondary effluents: coagulation, chemical precipitation with ferric chloride, and neutral Fenton reaction. Coagulation showed limited efficiency, with removal rates of less than 50% for several compounds; in particular, NPX and IBU achieved removal rates of 22% and 19%, respectively. Chemical precipitation enabled the near-complete removal of some compounds, such as TC, PCM, and COT, from the effluent of the Guanajuato WWTP, as well as 100% removal of MTP from the effluent of the Valle de Bravo WWTP. In comparison, the neutral Fenton reaction achieved removal rates of up to 100% for some ECs and improved water quality with respect to organic matter and nutrients; therefore, the authors proposed it as a viable polishing alternative for secondary effluents [31].

International studies have also shown that removal efficiency depends on the type of treatment and the compound being evaluated. At five wastewater treatment plants (WWTPs) in China that discharge effluent into the Yangtze River, the presence of PCM, IBU, NPX, DCF, BEZ, and ATP at various concentrations was reported. A comparison of treatment processes indicated that the flocculation and sand filtration stages were effective at removing NPX and BEZ, whereas PCM and IBU were almost completely removed by biological treatment. Meanwhile, ATP and DCF were primarily removed through ultraviolet (UV) disinfection [58].

A similar trend was observed in Korea, where 58 PhACs were analyzed in the influents and effluents of 13 WWTPs. The highest concentrations in the influents were found for PCM, CAF, ASA, NPX, and IBU, with removal rates of 94–100%. However, iopamidol, cimetidine, CAF, diphenhydramine, and CBZ persisted mainly in the effluents, confirming that some compounds are not completely removed by conventional treatment processes [59].

Some PhACs may also persist after biological treatment and UV disinfection. In Portugal, Afonso et al. analyzed two WWTPs: the first operated with two treatment lines, one based on a trickling filter and the other on activated sludge, both followed by UV disinfection and an artificial maturation pond with a retention time of approximately 48 h. The second WWTP employed biological treatment using oxidation ditches with aerators, followed by UV disinfection, before discharging the effluent into the Ria Formosa coastal lagoon. Fourteen PhACs and two metabolites were detected in the effluents from both WWTPs and in the maturation lagoon, including fluoxetine, venlafaxine, SMX, TMP, ATE, clofibric acid, CAF, NPX, KTP, PCM, DCF, SA, CBZ, and IBU, as well as the metabolites 10,11-epoxycarbamazepine and 1-hydroxy-ibuprofen. The highest annual concentrations were for IBU, followed by CAF and DCF. However, this study did not report removal efficiencies; therefore, its results should be interpreted as evidence of residual persistence in treated effluents and not as a comparative evaluation of treatment performance [60].

Overall, the reviewed studies show that the efficiency of EC removal in wastewater treatment plants depends on both the configuration of the treatment system and the physicochemical properties of each compound. Biological treatments can achieve high removal rates for readily biodegradable compounds, such as PCM, IBU, and CAF; however, they are less efficient against persistent compounds, including CBZ, DCF, SMX, MTP, certain antibiotics, psychotropic drugs, and metabolites. This limitation is significant because, even though concentrations decrease during treatment, effluents can continue to act as point sources of chemical pollutants into rivers, lagoons, and coastal systems. In the case of Mexico, available evidence indicates that conventional wastewater treatment plants were not designed to remove these contaminants and that incorporating advanced processes—such as the Fenton reaction, ozonation, UV/H_2_O_2_, activated carbon, or combined treatments—could substantially improve effluent quality. Therefore, it is necessary to strengthen monitoring of multiple contaminants in influent and effluent streams, evaluate transformation products, and establish differentiated treatment strategies based on the type of contaminant, the receiving environment, and the associated ecological risk.

## 8. Emerging Contaminants in Mexican Environmental Matrices

**Figure 6:**
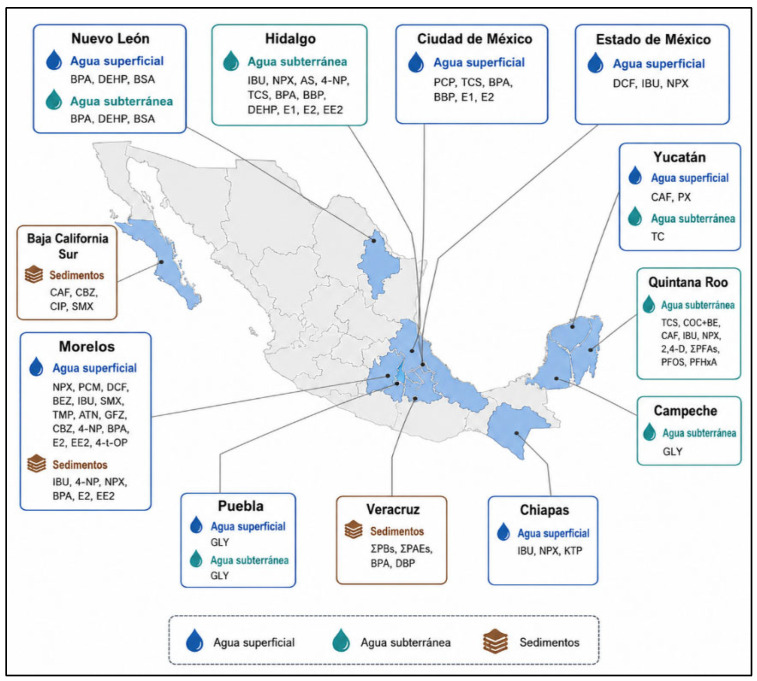
Spatial distribution of emerging contaminants by environmental matrix in Mexico.

Mexico has 32 states; however, the studies compiled in Table 3, Table 4 and Table 5 focus solely on 12 states, which account for 37.5% of the total. Consequently, 62.5% of the states lack reports on the matrices considered in this review. This indicates limited and heterogeneous geographic coverage, as shown in Figure 6. Furthermore, no state has reported research on all three environmental matrices, highlighting a significant geographic gap in the national monitoring of emerging contaminants.

Based on the collected site-matrix records, EC monitoring has focused primarily on surface water, which accounts for 46.9% of the records, followed by groundwater at 40.6%. In contrast, sediments account for only 12.5%, reflecting the fact that this matrix has been the least studied. This limitation is significant because sediments can act as reservoirs for persistent contaminants, especially hydrophobic compounds such as phthalates, bisphenols, and alkylphenols. Furthermore, under conditions of resuspension or physicochemical changes, sediments can act as secondary sources of contamination into the water column.

A comparative analysis of the findings summarized in Table 3, Table 4 and Table 5 reveals that the ECs reported in the three environmental matrices studied—surface water, groundwater, and sediments—correspond primarily to the NSAIDs IBU and NPX, the EDCs BPA and 4-NP, the hormones E2 and EE2, and the stimulant CAF. The presence of these compounds in all three matrices suggests greater environmental prevalence, greater mobility between compartments, or greater analytical focus on these contaminants in studies conducted in Mexico.

On the other hand, compounds of industrial origin and those associated with consumer products—such as BSA, DEHP, and BPA—as well as the pesticide GLY, have been reported in both surface water and groundwater. This distribution suggests a possible transfer from surface sources to the subsurface, particularly in areas influenced by urban, industrial, or agricultural activities. In the case of GLY, its presence in both matrices is primarily associated with agricultural activities and infiltration processes.

In contrast, several contaminants are reported in a single matrix, such as PCM, TMP, ATE, BEZ, GFZ, CIP, PCP, PX, SA, COC+BE, 2,4-D, TC, ΣPFAS, PFOS/PFHxA, ΣBPs, ΣPAEs, and DBP. This does not necessarily imply their absence in other matrices; rather, it reflects the limited spatial and analytical coverage available in Mexico, as well as a possible bias toward certain groups of compounds, environmental matrices, and study sites.

Finally, the highest concentrations recorded were for BSA and DEHP in Nuevo León, particularly in the Santa Catarina River and its groundwater, followed by EE2, BPA, E2, and 4-NP in the Apatlaco River basin in Morelos. The greater representation of Morelos should be interpreted as a geographic and publication bias, rather than as evidence that this state is necessarily more polluted than other regions of Mexico. These results show that the most common contaminants do not always reach the highest concentrations. Therefore, monitoring programs should prioritize both ECs that recur across different matrices and those that, although reported less frequently, reach high concentrations at specific sites.

## 9. Conclusions

This study shows that research on the presence of ECs in Mexican environmental matrices remains limited and concentrated in only a few regions of the country. The most common compounds include PhACs, EDCs, personal care products, phthalates, bisphenols, xanthines, illicit drugs, and industrial compounds.

Systems affected by wastewater discharges exhibit the greatest diversity of ECs, especially in areas with high population density, industrial activity, or tourism pressure. In surface waters, IBU, NPX, DCF, BPA, BSA, 4-NP, E2, EE2, DEHP, CAF, and its metabolite PX are particularly prevalent. In groundwater, IBU, NPX, TCS, BPA, BBP, DEHP, E1, E2, EE2, CAF, 2,4-D, TC, cocaine and its metabolite benzoylecgonine, as well as SA, a metabolite of ASA, have been reported. These findings indicate that some ECs can migrate into aquifers via infiltration and recharge.

Sediments are a key matrix for understanding the environmental fate of ECs, as they can act as both sinks and secondary sources of contamination. PhACs, EDCs, bisphenols, and phthalates have been detected in Mexican sediments; the latter are present at the highest concentrations, suggesting a greater affinity for organic matter and sedimentary particles.

Conventional wastewater treatment plants (WWTPs) do not completely remove ECs. Although some biodegradable compounds, such as PCM, IBU, and CAF, may exhibit high removal rates, others, such as CBZ, DCF, SMX, and MTP, as well as antibiotics, psychotropic drugs, and metabolites, persist in effluents. Consequently, WWTPs can act as point sources discharging ECs into receiving waters.

In Mexico, the lack of systematic monitoring and specific regulations limits a comprehensive assessment of the environmental risk associated with ECs. It is necessary to expand studies on under-evaluated matrices, such as sediments, aquifers, and hospital effluents, and to implement advanced treatment strategies, environmental monitoring, and discharge management to reduce the entry of these contaminants into aquatic ecosystems.

## Figures and Tables

**Table 1:** Pollutants and their classification.

Classification	Substance	Abbreviation
Nonsteroidal Anti-Inflammatory Drugs (NSAIDs)	Acetylsalicylic acid	ASA
	Ibuprofen	IBU
	Diclofenac	DCF
	Naproxen	NPX
	Ketoprofen	KTP
	Ketorolac	KET
Pain relievers	Paracetamol/ Acetaminophen	PCM
Anticonvulsants	Carbamazepine	CBZ
Antibiotics	Trimethoprim	TMP
	Penicillin G	PEN G
	Penicillin V	PEN V
	Amoxicillin	AMP
	Sulfamethoxazole	SMX
	Tetracycline	TC
	Ciprofloxacin	CIP
	Clarithromycin	CLA
	Ofloxacin	OFX
	Norfloxacin	NFX
Antihypertensives/β-blockers	Valsartan	VAL
	Atenolol	ATE
	Metoprolol	MTP
	Propranolol	PRO
Antidiabetics	Metformin	MTF
	Glibenclamide	GLB
Antihistamines	Diphenhydramine	DPH
Bronchodilators	Albuterol	ALB
Bisphenols	Bisphenol S	BPS
	Bisphenol F	BPF
	Bisphenol AF	BPAF
	Bisphenol B	BPB
Lipid-lowering drugs	Bezafibrate	BEZ
	Gemfibrozil	GFZ
Psychotropic drugs	Lormetazepam	LOR
	Diazepam	DZP
	Alprazolam	ALP
	Fluoxetine	FLX
	Sertraline	SRT
	Meprobamate	MEP
Endocrine disruptors	Bisphenol A	BPA
	4-Nonylphenol	4-NP
	4-tert-octylphenol	4-t-OP
Illegal drugs	Cocaine	COC
Phthalates/plasticizers	Dibutyl phthalate	DBP
	Benzyl butyl phthalate	BBP
	Di(2-ethylhexyl) phthalate	DEHP
	Dimethyl phthalate	DMP
	Diethyl phthalate	DEP
	Dioctyl phthalate	DOP
Hormones	Estrone	E1
	Estradiol, 17-β-estradiol	E2
	Ethinylestradiol, 17-α-ethinylestradiol	EE2
	Androsterone	ADT
Stimulants/ Xanthines	Caffeine	CAF
	Theophylline	THEO
Personal care products	Triclosan	TCS
Pesticides	Pentachlorophenol	PCP
	2,4-Dichlorophenoxyacetic acid	2,4-D
	Glyphosate	GLY
Industrial	Polyethylene glycol	PEG
	Benzene sulfonamide	BSA
	Chlorophenol	CP
Perfluoroalkyl and polyfluoroalkyl substances (PFAS)	Perfluorooctanesulfonic acid	PFOS
	Perfluorohexanoic acid	PFHxA

**Table 2:** Metabolites and transformation products of emerging contaminants reported in aquatic matrices.

Original Compound	Metabolite/Transformation Product	Abbreviation
Acetylsalicylic acid	Salicylic acid	SA
Caffeine	Paraxanthine/1,7-dimethylxanthine	PX
Cocaine	Benzoylecgonine	BE
Erythromycin	Erythromycin-H_2_O	ERY-H_2_O
Nicotine	Cotinine	COT

**Table 3:** Emerging contaminants detected in inland and coastal surface waters in Mexico.

Body of Water	Contaminants	Reference
Xochimilco Wetlands, Mexico	Pentachlorophenol (PCP), TCS, BPA, BBP, E1, and E2 detected; no quantification reported	[22]
Coatán River, Tapachula, Chiapas	31,300 ± 23,040 ng/L IBU 75,840 ± 18,820 ng/L NPX 8370 ± 6040 ng/L KTP	[21]
Texcuyuapan River, Tapachula, Chiapas	6810 ± 640 ng/L IBU 48,970 ± 31,590 ng/L NPX 8390 ± 6750 ng/L KTP	[21]
Apatlaco River, Cuernavaca, Morelos	732–4880 ng/L NPX, 354–4460 ng/L PCM, 258–1398 ng/L DCF, 286–2100 ng/L Bezafibrate (BEZ), 184–1106 ng/L IBU, 76–722 ng/L SMX, 34–120 ng/L trimethoprim (TMP), 4–32 ng/L atenolol (ATE), 9–368 ng/L GFZ and 8–276 ng/L CBZ	[26]
Madín Reservoir, Mexico	0.29–0.46 ng/L DCF 3.78–5.12 ng/L IBU 0.10–0.32 ng/L NPX	[25]
Apatlaco River Basin (Cuernavaca, Temixco, and Jiutepec), Morelos	Maximum values 85,500 ng/L 4-NP, 174,600 ng/L BPA, 103,600 ng/L E2, and 624,300 ng/L EE2	[35]
Cuautla River, Morelos	22.46 ± 30.17 ng/L BPA, 11.24 ± 11.76 ng/L 4-tert-octylphenol (4-t-OP), 7.53 ± 14.88 ng/L 4-NP, 2.37 ± 4.36 ng/L EE2, 0.97 ± 1.82 ng/L E2	[20]
Surface waters of the municipality of Tenampulco, Puebla	330–3280 ng/L GLY	[27]
Apatlaco River Basin (Cuernavaca, Temixco, and Jiutepec), Morelos	17.74 ± 23.19 ng/L IBU, 5.94 ± 5.07 ng/L 4-NP, 50.90 ± 45.94 ng/L NPX, 23.22 ± 24.74 ng/L BPA, 2.86 ± 1.89 ng/L E2 and 3.24 ± 7.17 ng/L EE2	[24]
Celestún Coastal Lagoon, Yucatán	96.8–688.3 ng/L CAF, 26–814.5 ng/L PX	[23]
Chelem Coastal Lagoon, Yucatán	113.2–2385.7 ng/L CAF, 14.5–301.3 ng/L PX	[23]
Dzilam de Bravo Coastal Lagoon, Yucatán	46.3–98 ng/L CAF, 8.7–44.4 ng/L PX	[23]
Ría Lagartos Coastal Lagoon, Yucatán	21.8–157.3 ng/L CAF, 37.6 ng/L PX	[23]
Surface waters of the Santa Catarina River, Monterrey, Nuevo León, Mexico (low-flow season)	38,000 ± 7000 ng/L BPA, 127,000 ± 8000 to 707,000 ± 35,000 ng/L DEHP, 691,000 ± 55,000 to 217,2000 ± 13,000 ng/L Benzenesulfonamide (BSA)	[36]
Surface waters of the Santa Catarina River, Monterrey, Nuevo León, Mexico (rainy season)	20,000 ± 11,000 to 67,000 ± 7000 ng/L BPA, 396,000 ± 30,000 to 4,480,000 ± 12000 ng/L DEHP, 538,000 ± 8000 to 5,300,000 ± 37,000 ng/L BSA	[36]

**Table 4:** Emerging contaminants detected in Mexico’s aquifers and groundwater.

Aquifer/Groundwater	Pollutants	Reference
Cerro Colorado Springs, Tula, Hidalgo	0.8–2.2 ng/L IBU, 0.8–0.9 ng/L NPX, 7.8–9.6 ng/L SA, 1.8–8.0 ng/L 4-NP, 1.12–1.30 ng/L TCS, 0.40–0.41 ng/L BPA, 1.7–2.0 ng/L BBP, 6.6–25 ng/L DEHP, 0.16–0.17 ng/L E1, 0.01–0.02 ng/L E2, 0–0.06 ng/L EE2	[45]
Riviera Maya Aquifer, Quintana Roo (Tulum)	0.81 ng/L TCS, 1.87 ng/L COC + BE	[19]
Riviera Maya Aquifer, Quintana Roo (Puerto Aventuras)	12.50 ng/L CAF, 4.27 ng/L IBU, 3.19 ng/L NPX, 2.39 ng/L 2,4-D	[19]
Kikil Cenote, Yucatán	Tetracycline (TC) detected; no concentration reported	[46]
Chach Mool Cenote, Yucatán	TC detected; no concentration reported	[46]
Ich-Ek Well, Campeche	1410–1420 ng/L GLY	[43]
San Francisco Suc-Tuc Well, Campeche	1250 ng/L GLY	[43]
Xmaben Well, Campeche	1270 ng/L GLY	[43]
Sahcabchén, Cancabchén, Bolonchén, and Pakchén wells in the municipality of Hopelchén, Campeche	Presence of GLY	[43]
Wells in the municipality of Tenampulco, Puebla	260–3170 ng/L GLY	[27]
Groundwater from the Santa Catarina River, Monterrey, Nuevo León, Mexico (low-flow season)	30,000 ± 3000 to 35,000 ± 4000 ng/L BPA, 482,000 ± 22,000 to 789,000 ± 29,000 ng/L DEHP, 4,034,000 ± 15,000 to 9,575,000 ± 284,000 ng/L BSA	[36]
Groundwater from the Santa Catarina River, Monterrey, Nuevo León, Mexico (rainy season)	33,000 ± 4000 to 73,000 ± 4000 ng/L BPA, 274,000 ± 7000 to 932,000 ± 34,000 ng/L DEHP, 274,000 ± 16,000 to 11,743,000 ± 53,000 ng/L BSA	[36]
10 cenotes between Tulum and Playa del Carmen (Mayan Aquifer)	0.68–10.71 ng/L of ΣPFAS; PFOS and PFHxA were detected.	[44]

**Table 5:** Emerging contaminants detected in sediments from water bodies in Mexico.

Sediments	Pollutants	Reference
Sediments from the Apatlaco River (Cuernavaca, Temixco, and Jiutepec), Morelos, Mexico	0.48 ± 0.28 ng/g IBU, 0.22 ± 0.30 ng/g 4-NP, 0.31 ± 0.41 ng/g NPX, 1.04 ± 1.67 ng/g BPA, 0.40 ± 0.40 ng/g E2 and 0.09 ± 0.21 ng/g EE2	[24]
Surface sediments of La Paz Lagoon, Baja California Sur	7.33 to 37.68 ng/g CAF, 1.13 to 1.86 ng/g CBZ, 4.09 to 31.04 ng/g CIP, 5.69 to 44.77 ng/g SMX	[29]
Surface sediments of the SAV	7.0 × 10^−2^ to 1.35 ng/g ΣBPs, 0.18 to 4.59 × 10^3^ ng/g ΣPAEs BPA 0.66 ng/g BPA, 2.58 × 10^3^ ng/g DBP	[28]
SAV Trap Sediments	0.12 to 3.17 × 10^3^ ng/g ΣPAEs	[28]

**Table 6:** Emerging contaminants detected in Mexican wastewater treatment plants.

WWTP/Sampling Point	Pollutants	Reference
Inflow to the Ciudad Universitaria, Coyoacán, and Cerro de la Estrella WWTPs	200–2800 ng/L IBU, 70–5000 ng/L CP, 800–10,000 ng/L TCS, 200–4000 ng/L BPA, 2800–54,000 ng/L NPX	[30]
Effluent from the Ciudad Universitaria, Coyoacán, and Cerro de la Estrella WWTPs	60–340 ng/L IBU, 200–1400 ng/L CP, 80–300 ng/L TCS, 20–400 ng/L BPA, 100–800 ng/L NPX	[30]
Inflow to the Guanajuato WWTP	0–94,600 ng/L MTF, PCM, CAF, PX, NPX, theophylline (THEO), IBU	[31]
Inflow to the Valle de Bravo WWTP, State of Mexico	0–32,100 ng/L MTF, PCM, CAF, PX, THEO, NPX, androsterone (ADT)	
Effluent from the Atapaneo WWTP, Morelia (discharged into the Rio Grande)	135,000 ng/L polyethylene glycol (PEG), 64,900 ng/L TC, 15,500 ng/L amoxicillin (AMP), 12,200 ng/L lormetazepam (LOR)	[32]

**Table 7:** EC removal efficiencies in WWTPs.

WWTP	Treatment	Removal	Reference
Guanajuato, Mexico. 55 ECs during the rainy season and 53 ECs during the dry season	Oxidation ditches with simultaneous nitrification- denitrification + UV disinfection	20–22% overall EC removal efficiencies	[31]
Valle de Bravo, State of Mexico. 41 ECs during the rainy season and 44 ECs during the dry season	Anaerobic/ anoxic/aerobic reactors + combined disinfection with ClO_2_ and UV light	66–80% overall EC removal efficiencies	[31]
Atapaneo, Morelia, Michoacán, Mexico	Screening, primary sedimentation, biological filters, biological treatment with activated sludge, secondary sedimentation, chlorination, recirculation, and sludge treatment	10.7% for hormones and steroids; 13.6% for antibiotics; 26.6% for anxiolytics; 27.7% for anesthetics; 30.8% for antihistamines; 33.3% for drugs of abuse; and 63.9% for analgesics	[32]
Montreal, Canada	Screening and flocculation with alum and FeCl_3_ at concentrations of 10 mg/L and 10–20 mg/L, respectively	<30% for IBU, DCF, KTP, NPX, SA, TCS, CBZ, fenoprofen, clofibric acid, and the metabolite 2-OH-IBU	[57]
Granby, Canada	Screen and sand filtration, followed by secondary treatment with aerobic activated sludge	>93% for SA and IBU; >50% for NPX, TCS, and the 2-OH-IBU metabolite	[57]
Plant A, China	Anaerobic– anoxic–aerobic + secondary clarification + inclined plate clarifier	100% for PCM, IBU, BEZ, and nifedipine; 97% for antipyrine (ATP); 73% for NPX; and 6% for DCF	[58]
Plant B, China	Sequential Batch Reactor (SBR) + Fenton + Sedimentation	100% for DCF; negative removal of ATP (−11%) and BEZ (−23%)	[58]
Plant C, China	Anaerobic– anoxic–aerobic + secondary clarification + inclined plate clarifier	100% for ATP; 99% for DCF; 33% for 4-chlorobenzoic acid (4-CBA, a transformation product of BEZ); 16% for clofibric acid; and negative removal of BEZ (−3%)	[58]
Plant E, China	Anaerobic–anoxic–aerobic + secondary clarification + sand filter	100% for PCM, IBU, and nifedipine; 82% for 4-CBA; 71% for BEZ; negative removal of ATP (−9%) and DCF (−1463%)	[58]
WWTPs 1 and 2, Korea	Biological process or Hanwha Dynamic Flow (HDF) system	Average removal rate >97% for CAF, PCM, ASA, NPX, and IBU. No removal was observed for chlorpheniramine, DCF, iopamidol, and propranolol	[59]
WWTPs 12 and 13, Korea	KSBNR biological nutrient removal system and continuous-feed sequential batch reactor (CF-SBR)	Removal >90% for PCM, ASA, IBU, cephalexin, cefradine, and androstenedione; 50–90% for CAF, NPX, quetiapine, and TMP. ATE, cimetidine, SMX, iopamidol, and DCF were not removed	[59]

## Data Availability

Data are contained within the article.

## Abbreviations

BOD_5_	Biochemical oxygen demand
BPs	Bisphenols
CF-SBR	Continuous-feed sequencing batch reactor
ClO_2_	Chlorine dioxide
COD	Chemical oxygen demand
CONAGUA	National Water Commission
EC	Emerging contaminant
ECs	Emerging contaminants
EC50	Median effective concentration
EDCs	Endocrine-disrupting compounds
ELISA	Enzyme-linked immunosorbent assay
ESI-MS-TOF	Electrospray ionization mass spectrometry-time of flight
FTIR	Fourier-transform infrared spectroscopy
GC-MS	Gas chromatography-mass spectrometry
GC-MS/MS	Gas chromatography-tandem mass spectrometry
GRULAC	Latin America and the Caribbean Group
HDF	Hanwha Dynamic Flow
HLB	Hydrophilic–lipophilic balance
HPLC-MS/MS	High-performance liquid chromatography-tandem mass spectrometry
IC20	20% inhibitory concentration
IMSS	Mexican Social Security Institute
KSBNR	Kist Shinwon Biological Nutrient Removal
LC50	Median lethal concentration
LC-MS/MS	Liquid chromatography-tandem mass spectrometry
NOMs	Mexican Official Standards
NSAIDs	Nonsteroidal anti-inflammatory drugs
PAEs	Phthalic acid esters
PAHs	Polycyclic aromatic hydrocarbons
PFAS	Perfluoroalkyl and polyfluoroalkyl substances
PhACs	Pharmaceutically active compounds
POCIS	Polar organic chemical integrative sampler
PRISMA-ScR	Preferred Reporting Items for Systematic Reviews and Meta-Analyses extension for Scoping Reviews
RQ	Risk quotient
SAV	Veracruz Reef System
SBR	Sequencing batch reactor
SINA	National Water Information System
SPE	Solid-phase extraction
SPMD	Semi-permeable membrane device
SPME	Solid-phase microextraction
ΣBPs	Sum of bisphenols
ΣPAEs	Sum of phthalic acid esters
ΣPFAS	Sum of perfluoroalkyl and polyfluoroalkyl substances
TDS	Total dissolved solids
TN	Total nitrogen
TOC	Total organic carbon
TP	Total phosphorus
TSS	Total suspended solids
UHPLC	Ultra-high-performance liquid chromatography
UHPLC-MS/MS	Ultra-high-performance liquid chromatography-tandem mass spectrometry
UV	Ultraviolet radiation
UV/H_2_O_2_	Ultraviolet/hydrogen peroxide
WWTP	Wastewater treatment plant
WWTPs	Wastewater treatment plants

## References

[1] Mehariya S., Das P., Thaher M.I., Quadir M.A., Khan S., Sayadi S., Hawari A.H., Verma P., Bhatia S.K., Karthikeyan O. (2024). Microalgae: A Potential Bioagent for Treatment of Emerging Contaminants from Domestic Wastewater. Chemosphere.

[2] Sandoval J.A., Morales-Granados M.A., Rubio D. (2020). Breve Revisión del Uso de Microalgas para la Remoción de Contaminantes Emergentes en Aguas Residuales. Gest. Ambiente.

[3] Moraga V., Gaete H. (2024). Evaluación Ecotoxicológica de las Interacciones entre Paracetamol e Ibuprofeno sobre la Microalga de Agua Dulce *Pseudokirchneriella subcapitata*. Rev. Int. Contam. Ambient..

[4] Sánchez-Gonzales M., Alvariño L., Iannacone J. (2025). Riesgo Ecológico por Dos Productos Farmacéuticos, Diclofenaco e Ibuprofeno, en *Daphnia magna* (Pulga de Agua), *Paracheirodon innesi* (Pez Tetra Neón) y *Lemna gibba* (Lenteja de Agua). Rev. Investig. Vet. Perú.

[5] Cleuvers M. (2004). Mixture Toxicity of the Anti-Inflammatory Drugs Diclofenac, Ibuprofen, Naproxen, and Acetylsalicylic Acid. Ecotoxicol. Environ. Saf..

[6] Hernández-Tenorio R., Villanueva-Rodríguez M., Guzmán-Mar J.L., Hinojosa-Reyes L., Hernández-Ramírez A., Vigil-Castillo H.H. (2024). Priority List of Pharmaceutical Active Compounds in Aquatic Environments of Mexico Considering Their Occurrence, Environmental and Human Health Risks. Environ. Toxicol. Pharmacol..

[7] aus der Beek T., Weber F.A., Bergmann A., Hickmann S., Ebert I., Hein A., Küster A. (2016). Pharmaceuticals in the Environment—Global Occurrences and Perspectives. Environ. Toxicol. Chem..

[8] (2021). Que Establece los Límites Permisibles de Contaminantes en las Descargas de Aguas Residuales en Cuerpos Receptores Propiedad de la Nación.

[9] (1997). Que Establece los Límites Máximos Permisibles de Contaminantes en las Descargas de Aguas Residuales en Aguas y Bienes Nacionales.

[10] (2021). Límites Permisibles de la Calidad del Agua.

[11] (2003). Requisitos para la Recarga Artificial de Acuíferos con Agua Residual Tratada.

[12] (2007). Secretaría de Medio Ambiente y Recursos Naturales.

[13] U.S. Environmental Protection Agency Per- and Polyfluoroalkyl Substances (PFAS): Final PFAS National Primary Drinking Water Regulation. https://www.epa.gov/sdwa/and-polyfluoroalkyl-substances-pfas.

[14] European Union Directive 2008/105/EC of the European Parliament and of the Council. https://eur-lex.europa.eu/legal-content/EN/TXT/?uri=CELEX%3A02008L0105-20260510.

[15] European Union Directive 2006/118/EC of the European Parliament and of the Council. https://eur-lex.europa.eu/legal-content/EN/TXT/?uri=CELEX%3A02006L0118-20260510.

[16] CONAGUA Estándares de Calidad del Agua. https://www.gob.mx/conagua/es/articulos/indicadores-de-calidad-del-agua?idiom=es.

[17] CONAGUA SINA: Estadísticas de Calidad del Agua por Tipo de Cuerpo de Agua. https://sinav30.conagua.gob.mx:8080/SINA/.

[18] Tricco A.C., Lillie E., Zarin W., O’Brien K.K., Colquhoun H., Levac D., Moher D., Peters M.D.J., Horsley T., Weeks L. (2018). PRISMA Extension for Scoping Reviews (PRISMA-ScR): Checklist and Explanation. Ann. Intern. Med..

[19] Metcalfe C.D., Beddows P.A., Bouchot G.G., Metcalfe T.L., Li H., Van Lavieren H. (2011). Contaminants in the Coastal Karst Aquifer System along the Caribbean Coast of the Yucatan Peninsula, Mexico. Environ. Pollut..

[20] Calderón-Moreno G.M., Vergara-Sánchez J., Saldarriaga-Noreña H., García-Betancourt M.L., Domínguez-Patiño M.L., Moeller-Chávez G.E., Ronderos-Lara J.G., Arias-Montoya M.I., Montoya-Balbas I.J., Murillo-Tovar M.A. (2019). Occurrence and Risk Assessment of Steroidal Hormones and Phenolic Endocrine Disrupting Compounds in Surface Water in Cuautla River, Mexico. Water.

[21] Cruz-Esteban S., Cruz-López L., Malo E.A., Valle-Mora J., Infante-Matha D.M., Santiesteban-Hernández A., Gutiérrez Hernández R., Bello-Mendoza R. (2014). Presence of Anti-Inflammatory Drugs in Surface Waters of Tapachula Chiapas, Mexico. Rev. AIDIS Ing. Cienc. Ambient. Investig. Desarro. Pract..

[22] Díaz-Torres E., Gibson R., González-Farías F., Zarco-Arista A.E., Mazari-Hiriart M. (2013). Endocrine Disruptors in the Xochimilco Wetland, Mexico City. Water Air Soil Pollut..

[23] Martínez-Casales Y., León-Aguirre K., Lamas-Cosío E., Noreña-Barroso E., Herrera-Silveira J., Arcega-Cabrera F. (2022). Caffeine and Paraxanthine as Tracers of Anthropogenic Wastewater in Coastal Lagoons in Yucatan, Mexico. Bull. Environ. Contam. Toxicol..

[24] Ronderos-Lara J.G., Saldarriaga-Noreña H., Murillo-Tovar M.A., Alvarez L., Vergara-Sánchez J., Barba V., Guerrero-Alvarez J.A. (2022). Distribution and Estrogenic Risk of Alkylphenolic Compounds, Hormones and Drugs Contained in Water and Natural Surface Sediments, Morelos, Mexico. Separations.

[25] Pérez-Coyotl I., Martínez-Vieyra C., Galar-Martínez M., Gómez-Oliván L.M., García-Medina S., Islas-Flores H., Pérez-Pasten Borja R., Gasca-Pérez E., Novoa-Luna K.A., Dublán-García O. (2017). DNA Damage and Cytotoxicity Induced on Common Carp by Pollutants in Water from an Urban Reservoir. Madín Reservoir, a Case Study. Chemosphere.

[26] Rivera-Jaimes J.A., Postigo C., Melgoza-Alemán R.M., Aceña J., Barceló D., López de Alda M. (2018). Study of Pharmaceuticals in Surface and Wastewater from Cuernavaca, Morelos, Mexico: Occurrence and Environmental Risk Assessment. Sci. Total Environ..

[27] Reynoso E.C., Peña R.D., Reyes D., Chavarin-Pineda Y., Palchetti I., Torres E. (2020). Determination of Glyphosate in Water from a Rural Locality in México and Its Implications for the Population Based on Water Consumption and Use Habits. Int. J. Environ. Res. Public Health.

[28] Salazar-Remigio L., Ponce-Vélez G., Olivares-Rubio H.F., Amador-Muñoz O., Márquez-García A.Z., Ontiveros-Cuadras J.F. (2025). Bisphenol and Phthalate Levels, Sources, and Hazard Estimation in Sediments from a Reef System: First Study in the Southern Gulf of Mexico. Environ. Pollut..

[29] Becerra-Rueda O.F., Rodríguez-Figueroa G.M., Marmolejo-Rodríguez A.J., Aguíñiga-García S., Durán-Álvarez J.C. (2024). Pharmaceutical Residues in Sediments of a Coastal Lagoon in Northwest Mexico-Occurrence and Environmental Risk Assessment. J. Xenobiotics.

[30] Peña-Álvarez A., Castillo-Alanís A. (2015). Identificación y Cuantificación de Contaminantes Emergentes en Aguas Residuales por Microextracción en Fase Sólida-Cromatografía de Gases-Espectrometría de Masas (MEFS-CG-EM). TIP Rev. Espec. Cienc. Quim. Biol..

[31] Estrada-Arriaga E.B., Cortés-Muñoz J.E., González-Herrera A., Calderón-Mólgora C.G., Rivera-Huerta M.L., Ramírez-Camperos E., Montellano-Palacios L., Gelover-Santiago S.L., Pérez-Castrejón S., Cardoso-Vigueros L. (2016). Assessment of Full-Scale Biological Nutrient Removal Systems Upgraded with Physico-Chemical Processes for the Removal of Emerging Pollutants Present in Wastewaters from Mexico. Sci. Total Environ..

[32] Robledo Zacarías V.H., Velázquez Machuca M.A., Montañez Soto J.L., Pimentel Equihua J.L., Vallejo Cardona A.A., López Calvillo M.D., Venegas González J. (2017). Hidroquímica y Contaminantes Emergentes en Aguas Residuales Urbano Industriales de Morelia, Michoacán, México. Rev. Int. Contam. Ambient..

[33] Pérez-Alvarez I., Islas-Flores H., Gómez-Oliván L.M., Barceló D., López De Alda M., Pérez Solsona S., Sánchez-Aceves L., SanJuan-Reyes N., Galar-Martínez M. (2018). Determination of Metals and Pharmaceutical Compounds Released in Hospital Wastewater from Toluca, Mexico, and Evaluation of Their Toxic Impact. Environ. Pollut..

[34] Félix-Cañedo T.E., Durán-Álvarez J.C., Jiménez-Cisneros B. (2013). The Occurrence and Distribution of a Group of Organic Micropollutants in Mexico City’s Water Sources. Sci. Total Environ..

[35] Ronderos-Lara J.G., Saldarriaga-Noreña H., Murillo-Tovar M.A., Vergara-Sánchez J. (2018). Optimization and Application of a GC-MS Method for the Determination of Endocrine Disruptor Compounds in Natural Water. Separations.

[36] Correa de León G. (2024). Presencia y Cuantificación de Disruptores Endocrinos en el Río Santa Catarina en la Zona Metropolitana de Monterrey: Estudio del Transporte de Contaminantes en la Zona de Conexión Hidráulica de las Aguas Superficiales y Subterráneas. Ph.D. Thesis.

[37] Hossain M.K., Anu M.A., Islam F., Molla M.O.F., Jahan M.S., Asha S.M.A.A., Islam R. (2025). Global Relevant Concentration of Emerging Contaminants: Species Sensitivity Distributions and Ecological Risks to Aquatic Organisms—A Comprehensive Data Synthesis. Hyg. Environ. Health Adv..

[38] Sánchez M., Ruiz I., Soto M. (2022). The Potential of Constructed Wetland Systems and Photodegradation Processes for the Removal of Emerging Contaminants—A Review. Environments.

[39] Arguello-Pérez M.Á., Ramírez-Ayala E., Mendoza-Pérez J.A., Monroy-Mendieta M.M., Vázquez-Guevara M., Lezama-Cervantes C., Godínez-Domínguez E., Silva-Bátiz F.A., Tintos-Gómez A. (2020). Determination of the Bioaccumulative Potential Risk of Emerging Contaminants in Fish Muscle as an Environmental Quality Indicator in Coastal Lagoons of the Central Mexican Pacific. Water.

[40] Liu J., Wu J., Rong S., Xiong Y., Teng Y. (2022). Groundwater Vulnerability and Groundwater Contamination Risk in Karst Area of Southwest China. Sustainability.

[41] Durán Álvarez J.C. (2009). Cuantificación de Doce Contaminantes Emergentes, Provenientes del Agua Residual Empleada para Riego, en Suelos del Distrito de Riego 03, Tula, Hidalgo. Ph.D. Thesis.

[42] Durán-Álvarez J.C., Prado-Pano B., Jiménez-Cisneros B. (2012). Sorption and Desorption of Carbamazepine, Naproxen and Triclosan in a Soil Irrigated with Raw Wastewater: Estimation of the Sorption Parameters by Considering the Initial Mass of the Compounds in the Soil. Chemosphere.

[43] Rendón-Von Osten J., Dzul-Caamal R. (2017). Glyphosate Residues in Groundwater, Drinking Water and Urine of Subsistence Farmers from Intensive Agriculture Localities: A Survey in Hopelchén, Campeche, Mexico. Int. J. Environ. Res. Public Health.

[44] Kopczynski S., Nolen R., Hala D., Lases-Hernández F., Escobedo-Hinojosa W., Arcega-Cabrera F., Oceguera-Vargas I., Quigg A. (2025). Investigation of Anthropogenic and Emerging Contaminants in Sinkholes (Cenotes) of the Great Mayan Aquifer, Yucatán Peninsula. Arch. Environ. Contam. Toxicol..

[45] Gibson R., Becerril-Bravo E., Silva-Castro V., Jiménez B. (2007). Determination of Acidic Pharmaceuticals and Potential Endocrine Disrupting Compounds in Wastewaters and Spring Waters by Selective Elution and Analysis by Gas Chromatography-Mass Spectrometry. J. Chromatogr. A.

[46] Moralez-Pérez D. (2021). Cuantificación de Contaminantes y Análisis de Diversidad de Zooplancton en Agua Subterránea de la Península de Yucatán. Master’s Thesis.

[47] Chiaia-Hernández A.C., Casado-Martinez C., Lara-Martin P., Bucheli T.D. (2022). Sediments: Sink, Archive, and Source of Contaminants. Environ. Sci. Pollut. Res..

[48] Noreña-Barroso E., Gold-Bouchot G., Ceja-Moreno V. (2007). Temporal Variation of Persistent Organic Pollutant (POP) Residue Concentrations in Sediments from the Bay of Chetumal, Mexico. Bull. Environ. Contam. Toxicol..

[49] Ramírez-Elías M.A., Córdova-Quiroz A.V., Cerón-Bretón J.G., Cerón-Bretón R.M., Osten J.R., Cortés-Simón J.H. (2016). Dichloro-Diphenyl-Trichloroethane (DDT) and Endosulfan in Sediments of Sabancuy Lagoon, Campeche, Mexico. Open J. Ecol..

[50] Botello A.V., de la Lanza Espino G., Villanueva Fragoso S., Ponce Velez G. (2020). Pollution Issues in Coastal Lagoons in the Gulf of Mexico. Lagoon Environments Around the World-A Scientific Perspective.

[51] Castañeda-Chávez M.d.R., Lango-Reynoso F., Navarrete-Rodríguez G. (2018). Hexachlorocyclohexanes, Cyclodiene, Methoxychlor, and Heptachlor in Sediment of the Alvarado Lagoon System in Veracruz, Mexico. Sustainability.

[52] Briones-Venegas A., Ponce-Vélez G., Elías-García V.G., Botello A.V. (2023). Organochlorine Contaminants in Sediments and Factors Influencing Their Distribution in the Natural Marine Protected Area in the Gulf of Mexico. Chemosphere.

[53] Pizzini S., Giubilato E., Morabito E., Barbaro E., Bonetto A., Calgaro L., Feltracco M., Semenzin E., Vecchiato M., Zangrando R. (2024). Contaminants of Emerging Concern in Water and Sediment of the Venice Lagoon, Italy. Environ. Res..

[54] Baltazar E.A.E., Petia M.N., Gabriela M.C., Gabriela M.M., Norma R.S., Manuel S.Z. (2013). Presencia y Tratamiento de Compuestos Disruptores Endócrinos en Aguas Residuales de la Ciudad de México Empleando un Biorreactor con Membranas Sumergidas. Ing. Investig. Tecnol..

[55] Arguello-Pérez M.Á., Mendoza-Pérez J.A., Tintos-Gómez A., Ramírez-Ayala E., Godínez-Domínguez E., Silva-Bátiz F.d.A. (2019). Ecotoxicological Analysis of Emerging Contaminants from Wastewater Discharges in the Coastal Zone of Cihuatlán (Jalisco, Mexico). Water.

[56] Liberti D., Pinheiro F., Simões B., Varela J., Barreira L. (2024). Beyond Bioremediation: The Untapped Potential of Microalgae in Wastewater Treatment. Water.

[57] Gagnon C., Lajeunesse A. (2012). Low Removal of Acidic and Hydrophilic Pharmaceutical Products by Various Types of Municipal Wastewater Treatment Plants. J. Xenobiotics.

[58] Li Y., Niu X., Yao C., Yang W., Lu G. (2019). Distribution, Removal, and Risk Assessment of Pharmaceuticals and Their Metabolites in Five Sewage Plants. Int. J. Environ. Res. Public Health.

[59] Son D.J., Kim C.S., Lee J.H., Yoon J.K., Lee S.H., Jeong D.H. (2023). Occurrence Assessment of Pharmaceuticals in Various Sewage Treatment Plants and Effluent-Receiving Streams in Korea. Water.

[60] Afonso V., Rodrigues B., Borges R., Barros R., Bebianno M.J., Raposo S. (2025). The Potential of Native Microalgae Consortia to Remove Pharmaceutical Compounds Present in Treated Wastewater. J. Environ. Manag..

